# Postacute Sequelae of COVID-19 in Pediatric Patients Within the United States: A Scoping Review

**DOI:** 10.1016/j.ajmo.2024.100078

**Published:** 2024-09-26

**Authors:** Christine M. Miller, Carla Borre, Alex Green, Melissa Funaro, Carlos R Oliveira, Akiko Iwasaki

**Affiliations:** a Department of Pediatrics, Division of Infectious Diseases and Global Health, Yale University School of Medicine New Haven, New Haven, CT; b Harvey Cushing/John Hay Whitney Medical Library, Yale University, New Haven, CT; c Department of Immunobiology, Yale School of Medicine, New Haven, CT; d Howard Hughes Medical Institute, Chevy Chase, MD; e Center for Infection and Immunity, Yale School of Medicine, New Haven, CT

**Keywords:** Long COVID, Postacute sequelae of COVID-19 (PASC), Pediatric

## Abstract

A subset of children and adolescents experience recurrent or persistent symptoms following SARS-CoV-2 infection, known as postacute sequelae of COVID-19 (PASC), however, the clinical epidemiology within the United States (US) is not yet well understood. This scoping review aims to synthesize the clinical epidemiology of pediatric PASC in the US. A comprehensive literature search was conducted and databases were queried from inception until January 29, 2024. Studies including US children and adolescents <21 years old were considered. From 1028 studies identified, 29 met the inclusion criteria. Prevalence of PASC ranged from less than 1%-27%. Risk factors included older age, female sex, asthma, obesity, and severe initial infection. Common symptoms were dyspnea, fatigue, headaches, and chest pain. A multidisciplinary approach for diagnosis and management was common across studies. Most studies had a high risk of bias and were limited by a lack of standardized definitions and short follow-up duration. This review establishes a foundation for understanding pediatric PASC and highlights the critical need for continued research to optimize prevention and treatment strategies.

**Clinical Significance**

• Our scoping review offers the advantage of conducting a thorough mapping and synthesis of existing literature on Pediatric PASC in the United States, encompassing a diverse array of evidence, methodologies, and sources.
• By providing a comprehensive review, we identify knowledge gaps that can serve as valuable insight for policymakers, healthcare providers, and other key stakeholders involved in addressing pediatric PASC.
• A limitation of this scoping review lies in the diverse nature of the articles included, leading to variability in the quality of evidence assessed.
• Our reliance on published literature constrained our scope, ultimately limiting a comprehensive understanding of the epidemiology of pediatric PASC in the US.

Alt-text: Unlabelled box

## Introduction

Postacute infection syndromes following various infections have been well-documented across the past two centuries.^1^ Following epidemics, postacute infection syndromes manifests in overlapping symptoms in a subset of patients. In the early 1890s, after the H2N2 influenza pandemic, physicians identified “neurasthenia” or “influenza exhaustion,” marked by symptoms of fatigue, muscle and neuropathic pains, headaches, and postexertional malaise occurring weeks to months after acute illness.^2^ Following the H1N1 influenza pandemic of 1918, “Encephalitis lethargica” emerged, characterized by excessive sleepiness, fevers, headaches, and movement disorders, notably affecting children.^3^ In the mid-20th century postpoliomyelitis syndrome was recognized, characterized by progressive muscle weakness, fatigue, and myopathy occurring several decades after recovery from paralytic polio.^4^ Today, postacute sequelae of COVID-19 (PASC) remains poorly understood, particularly in children. Recent technological advances offer promise for improving the understanding, diagnosis, and management of these conditions. This study seeks to illuminate the clinical epidemiology of pediatric PASC in the United States (US), underscoring the importance of translational research in identifying biomarkers and developing targeted therapeutics.

Since 2019, SARS-CoV-2 has infected over 15 million children and counting.^5^ Although most of these acute infections do not result in significant morbidity or mortality, a subset of children and adolescents experience recurrent or persistent symptoms that last beyond the typical recovery period. This collection of postinfection symptoms is known as Postacute-Sequelae of COVID-19 (PASC) or long COVID. Now, more than 4 years since the start of the SARS-CoV-2 pandemic, there is accumulating evidence of PASC in children.

Like adult PASC, pediatric PASC can be debilitating and severely impact affected patient's lives. Unlike adult PASC however, the disruption to the child's education and life has serious developmental implications including school absenteeism, loss of involvement in sports and extracurricular activities, and interrupted socialization. Because of this, it is critical for us to gain a better understanding of pediatric PASC to both increase awareness and improve care.

Understanding aspects of patient perception, stigma of illness, and care specifically within the US is also important. As a resource-rich country with a complex and fragmented healthcare system, the framework in which patients are treated is highly variable. To best support the children in our community who are suffering from this illness, we need to have a better understanding of the clinical epidemiology.

In this scoping review, we perform a literature review on the PASC on pediatric patients less than 21 years old within the US. The purpose of our study is to describe the current epidemiology, symptoms, diagnostic approaches, and treatments for pediatric PASC. While other studies have made important contributions describing pediatric PASC, there is very limited data synthesizing pediatric PASC specifically in the US. Therefore, the aim of this scoping review is to synthesize the best available evidence pertaining to the clinical epidemiology of pediatric PASC in the US.

## Methods

### Literature Search

To identify relevant literature, we conducted a medical subject heading analysis and systematically searched MEDLINE, Embase, CINAHL, Web of Science, and Cochrane databases. Searches were limited to English language articles; Dates from inception to June 28, 2022, were imposed on the search. To maximize sensitivity, the formal search used controlled vocabulary terms and synonymous free-text words to capture the concepts of “long COVID” and “pediatrics.” An iterative process was used to translate and refine the searches with the support of an experienced medical librarian. The search strategy was peer-reviewed using the PRESS standard by a second librarian not associated with the project.^6^ An overview of the search strategy is included in Appendix 1. Search results were pooled, de-duplicated, and uploaded to Covidence for screening.^7^ Subsequent to the screening process, backward and forward citation chasing was conducted to identify additional relevant articles. Two investigators (CMM and AG) independently reviewed the titles, abstracts, and full text of the eligible articles that met the inclusion criteria. Conflicts were resolved through consensus with a third reviewer (CB). Prior to submission, an updated search was done on January 29, 2024, to assess for any updates, and five additional studies were included in this review as well as full-text publications of preprints and poster abstracts identified through our original COVIDENCE search.

### Data Synthesis

Severity of initial COVID-19 symptoms was tabulated into 2 categories: mild or moderate/severe based on the descriptions from the included studies and plotted as a percent total (Supplementary Figure 1). For symptom prevalence, all PASC symptoms described in relevant studies were compiled into a list along with the number of patients in each study that reported those symptoms and subsequently combined and plotted as a percentage out of the total number of pediatric patients described (Supplementary Figure 2). PASC symptom duration was reported from relevant articles as the mean range with a 95% confidence interval. All analyses were conducted using R (version 4.3.3) and Microsoft Excel (version 16.85). The rest of the evidence described was qualitatively synthesized; meta-analyses were not conducted due to heterogeneity in study populations, testing, and reported outcomes. A “best evidence” synthesis strategy was adopted, prioritizing the data that directly addressed our research questions and demonstrated methodological rigor. Our protocol was developed using the Joanna Briggs Institute framework and adhered to the PRISMA Extension for Scoping Reviews (PRISMA-ScR) guidelines.^8^

### Inclusion Criteria

Inclusion criteria were children and adolescents less than 21 years of age within the US who had previous laboratory-confirmed COVID-19 infection or close contact with a confirmed case and symptoms consistent with COVID-19. Additionally, the patients had to have met criteria for PASC based on the case definitions from either the World Health Organization or Center for Disease Control (Appendix 2).^9^^,^^10^

### Bias Assessment

Risk of bias was assessed in all cohort and cross-sectional studies using the Quality in Prognostic Studies tool.^11^ The Quality in Prognostic Studies tool assesses the risk of bias in six domains^1^: study participation,^12^ study attrition,^8^ prognostic factor measurement,^11^ outcome measurement,^13^ study confounding, and^14^ statistical analysis and reporting. Studies were determined to have a high risk of bias if more than three domains were classified as moderate risk, or if one domain was high risk of bias. For studies that were not at high risk of bias but had more than 2 domains of moderate risk of bias, they were determined to be moderate risk overall. All others were classified as low risk of bias.

## Results

**Figure 1:**
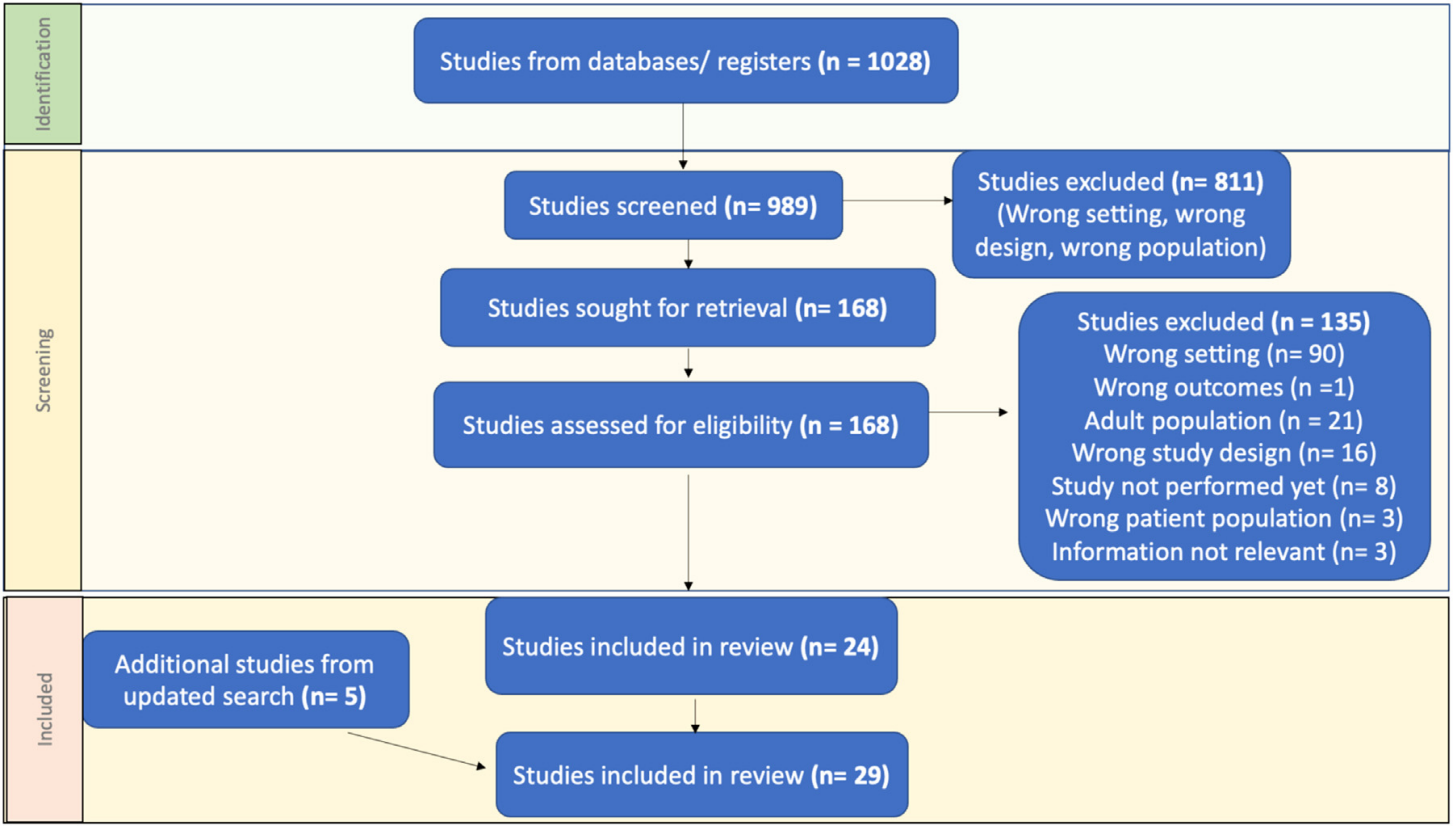
PRISMA graphic of literature review.

Out of the 1028 studies identified from databases/registers, 168 were screened for full review, 24 met inclusion criteria and 5 additional studies were identified through an updated search and included (Figure 1). Of the 29 studies that met inclusion criteria, 10 were single-center cohort studies, 4 were muti-center cohort studies, 3 cross-sectional studies, 3 systematic reviews (1 preprint), 4 case series, 1 case report, 1 consensus guidelines, and 3 educational/opinion pieces. Three of the above studies were poster abstracts (1 single-center cohort study, 1 multi-center cohort study, and 1 case series). Bias assessment was performed and the majority were at high risk of bias with the exception of two^15^^,^^16^ which were at moderate risk of bias.

### Epidemiology

The prevalence of pediatric PASC among the infected population ranged from less than 1% to nearly 27%.^13^^,^^14^ The largest and most compelling study was a multi-center retrospective electronic health record review which included 659,286 children and adolescents under age 21 and found the incidence of non-MISC PASC to be 3.7%.^15^

Most pediatric patients described with PASC symptoms were adolescents. There was a female as well as Caucasian ethnicity predominance. Asthma, atopy, allergic rhinitis, and obesity were among the most frequently described pre-existing conditions. Commonly described risk factors for developing PASC included increased age,^15^^,^^17^ female gender,^14^, ^15^, ^17^ asthma and atopy,^13^^,^^14^^,^^18^, ^19^, ^20^, ^21^, ^22^ obesity,^13^, ^14^, ^15^^,^^18^, ^19^, ^20^ and severe initial infection.^23^, ^24^ In a cohort study published by Leftin Dobkin et al,^25^ asthma prevalence was nearly five times higher in their study population with respiratory PASC compared to the prevalence in the general population, suggesting preexisting asthma as an important risk factor for PASC in children.

**Figure 2:**
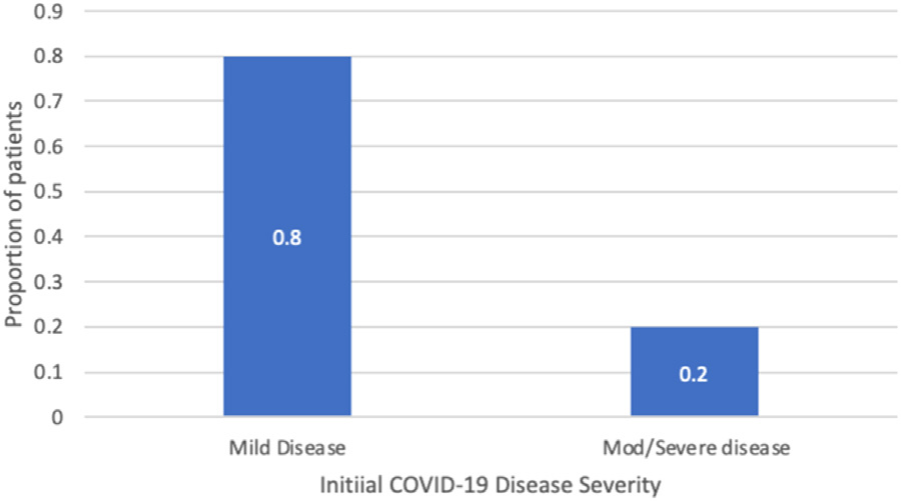
Initial COVID-19 illness severity, *n* 721.

Most (80%) patients in the articles included in this review had mild initial COVID-19 symptoms, such as fever, fatigue, cough, shortness of breath, loss of taste or smell, congestion, sore throat, headache, rash, arthralgia, and myalgia (Figure 2).

### Timing of Symptom Onset

**Figure 3:**
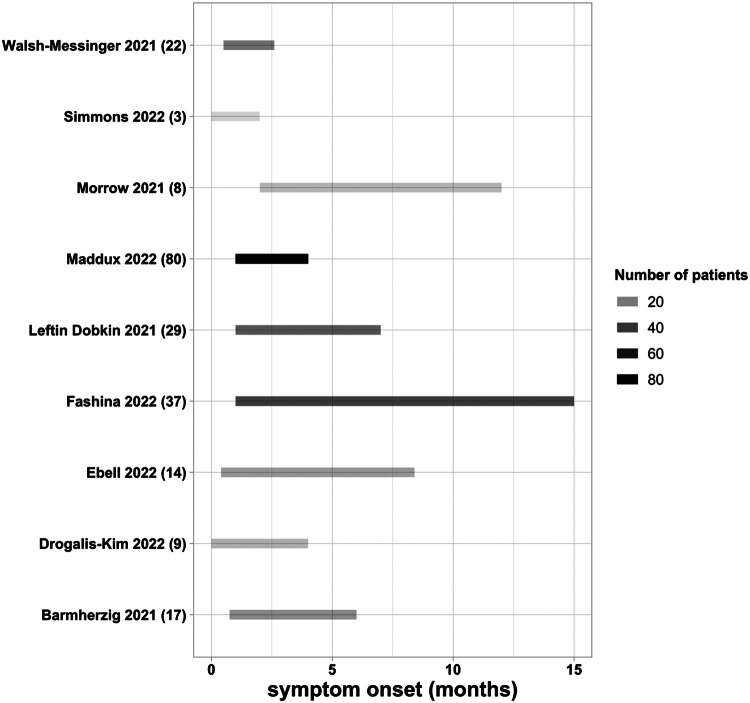
Range of pediatric PASC symptom onset following acute COVID-19. Number of patients included in parentheses.

The onset of PASC symptoms following acute COVID-19 illness ranged from less than 1-15 months (Figure 3). Three studies described patients who continued to have progressive symptoms following their initial COVID-19 infection.^26^, ^27^, ^28^ In a retrospective electronic health record review that included 9 different institutions, PASC patients were characterized as having symptoms 28-179 days following positive SARS-CoV-2 testing.^15^

### PASC Symptom Description

**Figure 4:**
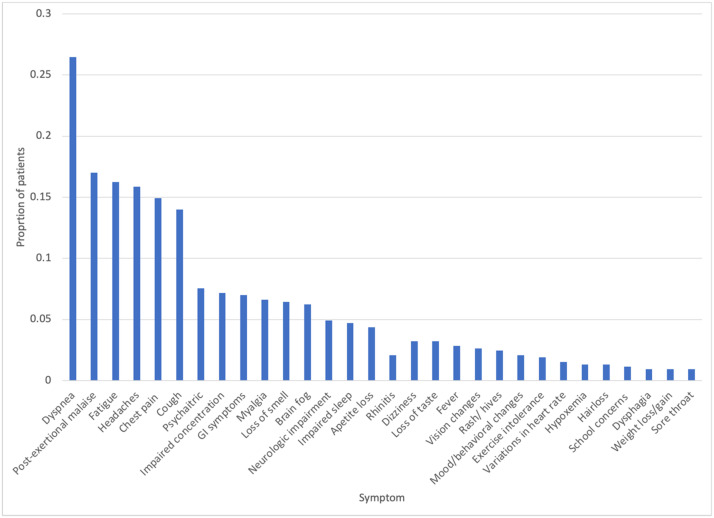
Pediatric PASC symptom prevalence, *n* 529.

The most frequently reported symptoms were dyspnea (26%),^14^^,^^18^, ^19^, ^20^, ^21^^,^^26^, ^29^, ^30^, ^31^, ^32^ postexertional malaise (17%),^18^^,^^20^^,^^22^^,^^33^ fatigue (16%)^13^^,^^14^^,^^18^^,^^21^^,^^22^^,^^26^, ^29^, ^30^, ^34^^,^^31^ headaches (16%)^13^, ^14^, ^16^, ^21^, ^22^, ^26^, ^29^, ^31^, ^33^, ^34^, ^35^, ^36^ and chest pain (15%)^13^^,^^20^, ^21^, ^22^^,^^26^^,^^29^^,^^30^ (Figure 4). Out of these studies, the largest was a single-center cohort study describing 82 patients seen at a pulmonary clinic for persistent pulmonary sequelae from COVID-19 infection.^20^

In a multi-center retrospective cohort study by Rao et al,^24^ the authors sought to identify PASC features through an electronic health record review. They identified 59,893 children who tested positive for SARS-CoV-2 and had a subsequent healthcare visit within the next 28-179 days and compared this group with a cohort of children with negative SARS-CoV-2 viral test findings during the same study period. They found the most common symptoms were loss of taste or smell (aHR 1.96; 95% CI 1.16-3.32), hair loss (aHR 1.58; 95% CI 1.24-2.01), and chest pain (aHR 1.52; 95% CI 1.38-1.68).^15^

Four articles include severe or life-threatening symptoms. One case study describes a 12-year-old female who developed progressive limb weakness and delirium, ultimately resulting in bed-ridden status following SARS-CoV-2 infection.^5^ Additionally, Guillain–Barre syndrome, adrenal insufficiency, central nervous system demyelination, encephalopathy, tic disorder, stroke, seizures, and paralysis were all rare PASC symptoms experienced.^19^^,^^37^^,^^38^

Several studies describe how PASC symptoms have impacted the children's day-to-day lives. One of them specifically mentions in their case series how four out of nine patients were athletes and had to stop playing their sport due to their symptoms.^26^ Limitations and decreased performance in school were also reported in several other studies due to the persistence of symptoms.^13^^,^^22^^,^^26^^,^^29^

### Diagnostic Approaches

Consensus guidelines have been published by the American Academy of Physical Medicine and Rehabilitation for the assessment and treatment of PASC in children and adolescents.^39^ These guidelines emphasize that PASC is a clinical diagnosis and therefore a thorough history and physical exam should be performed to ensure ruling out alternative diagnoses. We found in our review that most clinics focused on addressing patient's symptoms. Five studies involving patients with persistent neurologic symptoms used brain MRIs as part of their evaluation.^5^^,^^16^, ^36^, ^38^^,^^40^ One of these clinics also used electrodiagnostic nerve conduction studies to investigate limb weakness and sensory loss in their patient described.^5^ Three pulmonology clinics seeing patients with respiratory PASC symptoms used spirometry testing, plethysmography, and a 6-minute walk test for evaluations.^18^^,^^20^^,^^32^ Palacios et al^41^ and Nevid et al^32^ both also performed laryngoscopy on patients and found paradoxical vocal fold motion disorder and exercised-induced laryngeal obstruction, respectively. Two studies described patients who were seen in cardiology clinics for PASC symptoms where echocardiogram, EKG, Holter monitors, and Active Standing Tolerance Test were all used for diagnostic work-up.^26^^,^^42^ Active Standing Tolerance tests were the only evaluations to produce abnormal findings, however 5 out the 9 patients and 2 out of 7 patients seen were diagnosed with Postural Orthostatic Tachycardia Syndrome (POTS) in each study respectively.^26^^,^^42^

Morrow et al^43^ described their institution's development of a multi-disciplinary pediatric PASC clinic. To assess how the patient's symptoms were affecting their overall quality of life, the authors used the Pediatrics Quality of Life (PedsQL) survey and found children who were being evaluated for PASC had substantially lower quality of life scores in both the PedsQL Core survey and PedsQL Fatigue survey compared to healthy controls.^42^

### Treatment Approaches

Pediatric PASC treatments also tended to be geared toward specific symptom management or addressing any diagnoses found in discipline-specific work-ups. Patients who were diagnosed with POTS were treated with several different nonpharmacologic approaches (improved sleep hygiene, increased hydration, increased salt intake, exercise, and lower extremity compression garments) as well as several different medications (fludrocortisone, atenolol, midodrine).^26^^,^^42^ Once medication management was optimized, all five patients described (4 diagnosed with POTS, 1 did not meet criteria for POTS) had nearly complete symptom resolution within 1 week.^26^ Pulmonology clinics that diagnosed obstructive lung disorders treated patients with a bronchodilator, inhaled corticosteroid, or inhaled corticosteroid/long-acting beta-agonist combination.^18^^,^^20^^,^^32^ These three studies showed about 30% of patients responsive to inhaled bronchodilator therapy. Neurology clinics that were evaluating patients with headaches also trialed a combination of nonpharmacologic therapy, including improved sleep hygiene, adequate hydration, and limiting screen time, as well as pharmacologic management, including acetaminophen, aspirin, caffeine, ibuprofen and, in several cases, Triptans for rescue therapy.^35^^,^^36^ Barmherzig et al^15^ also described trials of nerve blocks for patients with severe headaches, however, this did not seem beneficial. In the development of a multi-disciplinary clinic described by Morrow et al,^43^ behavioral, neuropsychology, and physical therapy were all also included in their management approach, however did not indicate effectiveness. Acupuncture and vitamin supplementation (vitamin D, Co-enzyme Q, magnesium) were trialed with little observed benefit in several case series and a case report.^26^^,^^36^

## Discussion

To our knowledge, this is the first scoping review of pediatric PASC specifically describing epidemiologic observations seen within the US. Our study has revealed some similarities compared to adult data on PASC. For example, common symptoms described in adults include fatigue, brain fog, headaches, difficulty with memory, dizziness, difficulty sleeping, and dyspnea, which is very similar to what we describe here in pediatrics with PASC.^44^ However, in adults, fatigue is consistently described as the most frequently reported symptom, while this does not seem to be the case in pediatrics.^45^, ^46^, ^47^ In our pooled prevalence tabulation, dyspnea was the most prevalent symptoms (Figure 4). The association of female gender with pediatric PASC is another similarity to what has been seen in adult PASC.^47^, ^48^

Several studies reviewed highlighted severe or life-threatening complications arising from SARS-CoV-2 infection. While these conditions are categorized as post-COVID sequelae, the pathobiological mechanisms behind these entities are likely distinct from those of long COVID.

The studies in this review focus on addressing patient's symptoms as part of the work-up and ruling out alternative diagnoses. A study done at the University Hospital Essex found that out of 110 patients referred for long COVID evaluation, 21 patients were found to have an alternative diagnosis upon further investigation.^49^ Another recent international multi-center cross-sectional study found that most post-COVID multidisciplinary clinics were limited to treating symptoms with physical therapy and psychological support for their patients due to normal diagnostic testing in most cases.^50^

One of the biggest challenges in diagnosing PASC is the lack of objective measures to do so. In our review we describe how POTS has frequently been associated with PASC, however, PASC is a much more broad and heterogenous disorder. Several European studies have investigated other physiologic metrics as diagnostic tools and found that pediatric patients with PASC exhibited higher levels of autonomic dysautonomia via Holter EKG and Echocardiogram monitoring compared to controls^51^ as well as impaired cardiopulmonary functioning compared to controls.^52^ In another study, the authors evaluated the immunologic cellular phenotypes of pediatric PASC patients compared to convalescent controls and found persistent elevation in circulating T regulatory cells comparable to acute infection in male children.^53^ Although no precise biomarkers have been identified, these measures all take steps toward more objective diagnostics; an area greatly needed.

The largest prevalence study in this review was from Rao et al.^24^ under the NIH RECOVER initiative, where 9 different US-based healthcare systems underwent electronic health record review using ICD-10 codes to identify PASC within SARS-CoV-2 PCR positive patients vs PCR negative patients and found the prevalence of PASC to be 3.7%. In two recent studies in Denmark, a nationwide cross-sectional study was done on children aged 0-18 with confirmed SARS-CoV-2 PCR where a survey was either sent to the parent of the child (ages 0-14) or the child (ages 15-18) to assess long term symptoms and quality of life.^18^^,^^20^ Collectively these studies assessed 185,364 children and 121,572 adolescents respectively and found there to be a higher prevalence of longer symptoms in the case group compared to controls and prevalence varied based on age group, symptom type, and length of time since SARS-CoV-2 infection.

In our study, we found increased age, female sex, history of asthma/atopy obesity and severe initial SARS-CoV-2 infection to be associated with increased risk of PASC. Other studies outside of the US have had similar findings. Several large Italian studies and a Canadian study identified several risk factors for developing PASC including hospitalization, infection with a pre-Omicron variant, older age, allergic rhinitis, obesity, and previous respiratory disease associated with increased risk of PASC.^22^, ^54^

It's important to note that several published studies have found increased levels of type-2 immune factors in individuals with PASC compared to those who have recovered.^43^, ^44^ Given that conditions like asthma and atopic illnesses involve chronic type-2 inflammation, there's a pressing need for additional research in this area to shed light on the underlying mechanisms of PASC.

The studies included in our review did not have sufficient data to assess recovery rates, however, there have been other reports published generally indicating that most symptoms resolve by 12 months.^55^ In an Italian study 4% of their PASC cohort reported poor symptom recovery at 1-5 months, 1.3% at 6-9 months and 0.7% at 12 months following symptom onset.^56^ In a prospective cohort study done in Canada, 0.67% of patients who had tested positive for SARS-CoV-2 remained symptomatic at 12 months vs 0.16% of patients who had tested negative.^41^

Although the studies included in this review did not provide evidence on the role of vaccine effectiveness in the prevention of PASC, a recent publication indicates that there may be a modest benefit.^24^ This study noted a higher level of vaccine effectiveness against PASC development in adolescents (50%) vs 23.8% in children and at 6 months post symptom onset (61.4%) vs 10.6% at 18 months.^24^ In a scoping review consisting mostly of studies in adults, individuals who were unvaccinated for COVID-19 had more significant PASC symptoms compared to those who were vaccinated.^47^ More studies are needed to further clarify these findings.

## Limitations

There are several limitations in our scoping review. First, given the nature of the PASC as a clinical diagnosis and the lack of a standardized definition, there was significant heterogeneity. Additionally, nearly all the studies included were descriptive in nature and therefore had variety in their follow-up duration and methods of assessment. The information reviewed was noted to have an overall high level of bias in our assessment (Appendix 3).

## Conclusion

In this scoping review, we elucidate the characteristics and implications of PASC within the US. Our comprehensive analysis reveals that a notable subset of children and adolescents experience prolonged and diverse symptoms following COVID-19 infection. The review further highlights the critical role of a multidisciplinary approach in both diagnosis and treatment. It emphasizes the pressing need for ongoing research efforts to understand, prevent, diagnose, and effectively manage this developing public health concern more precisely. Additionally, it underscores the necessity for further pediatric translational research to identify biomarkers of this disease as well as clinical trials for potential therapies.

## Data Sharing Statement

All raw data for figures are listed in our supplementary data.

## CRediT authorship contribution statement

**Christine M. Miller:** Writing – review & editing, Writing – original draft, Formal analysis, Data curation, Conceptualization. **Carla Borre:** Writing – review & editing, Investigation. **Alex Green:** Writing – review & editing, Investigation. **Melissa Funaro:** Writing – review & editing, Methodology. **Carlos R Oliveira:** Writing – review & editing, Supervision, Methodology, Formal analysis, Conceptualization. **Akiko Iwasaki:** Writing – review & editing, Supervision, Conceptualization.

## Declaration of competing interest

The authors declare the following financial interests/personal relationships which may be considered as potential competing interests: A. I. co-founded RIGImmune, Xanadu Bio and PanV, and is a member of the Board of Directors of Roche Holding Ltd and Genentech. All other authors have no conflicts of interest and have signed below to indicate their agreement with this statement.

## Appendix Supplementary materials

Image, application 1

Image, application 2

Image, application 3
