# Supplementary material for: Postacute Sequelae of COVID-19 in Pediatric Patients Within the United States: A Scoping Review
Source: Am J Med Open. 2024 Sep 26;12:100078. doi: 10.1016/j.ajmo.2024.100078 (PMC11617896; doi:10.1016/j.ajmo.2024.100078)
Supplement: Supplementary file 1 [file mmc1.docx]

Appendix 1:

All Search Strategies

Ovid MEDLINE(R) ALL

| 1 | (long adj3 (covid* or ncov* or novel coronavirus or novel betacoronavirus or sars-ncov-2 or sars-cov-2)).mp. |
| --- | --- |
| 2 | (persist* adj3 (covid* or ncov* or novel coronavirus or novel betacoronavirus or sars-ncov-2 or sars-cov-2)).mp. |
| 3 | (chronic adj3 (covid* or ncov* or novel coronavirus or novel betacoronavirus or sars-ncov-2 or sars-cov-2)).mp. |
| 4 | ((long term or long-term or longterm) adj3 effect* adj3 (covid* or ncov* or novel coronavirus or novel betacoronavirus or sars-ncov-2 or sars-cov-2)).mp. |
| 5 | (sequela* adj3 (covid* or ncov* or novel coronavirus or novel betacoronavirus or sars-ncov-2 or sars-cov-2)).mp. |
| 6 | ((post acute or post-acute or postacute) adj3 (covid* or ncov* or novel coronavirus or novel betacoronavirus or sars-ncov-2 or sars-cov-2)).mp. |
| 7 | ((longhaul* or long haul* or long-haul*) adj3 (covid* or ncov* or novel coronavirus or novel betacoronavirus or sars-ncov-2 or sars-cov-2)).mp. |
| 8 | ((post-covid* or postcovid*) adj2 (syndrome or condition)).mp. |
| 9 | or/1-8 |
| 10 | exp pediatrics/ or exp child/ or exp adolescent/ or exp infant/ or (adolescence or adolescent or adolescents or babies or baby or child or children or childhood or infancy or infant or infants or juvenile or juveniles or kid or kids or newborn or new-born or newborns or new-borns or paediatric or paediatrics or pediatric or pediatrics or picu or perinatal or preadolescen* or pubescence or pubescent or school child or schoolchild or schoolchildren or teen or teens or teenager or teenagers or toddler or toddlers or youth or youths).mp. |
| 11 | 9 and 10 |
| 12 | limit 11 to english language |

Embase (Ovid)

| 1 | (long adj3 (covid* or ncov* or novel coronavirus or novel betacoronavirus or sars-ncov-2 or sars-cov-2)).mp. |
| --- | --- |
| 2 | (persist* adj3 (covid* or ncov* or novel coronavirus or novel betacoronavirus or sars-ncov-2 or sars-cov-2)).mp. |
| 3 | (chronic adj3 (covid* or ncov* or novel coronavirus or novel betacoronavirus or sars-ncov-2 or sars-cov-2)).mp. |
| 4 | ((long term or long-term or longterm) adj3 effect* adj3 (covid* or ncov* or novel coronavirus or novel betacoronavirus or sars-ncov-2 or sars-cov-2)).mp. |
| 5 | (sequela* adj3 (covid* or ncov* or novel coronavirus or novel betacoronavirus or sars-ncov-2 or sars-cov-2)).mp. |
| 6 | ((post acute or post-acute or postacute) adj3 (covid* or ncov* or novel coronavirus or novel betacoronavirus or sars-ncov-2 or sars-cov-2)).mp. |
| 7 | ((longhaul* or long haul* or long-haul*) adj3 (covid* or ncov* or novel coronavirus or novel betacoronavirus or sars-ncov-2 or sars-cov-2)).mp. |
| 8 | ((post-covid* or postcovid*) adj2 (syndrome or condition)).mp. |
| 9 | or/1-8 |
| 10 | exp pediatrics/ or exp child/ or exp adolescent/ or exp infant/ or (adolescence or adolescent or adolescents or babies or baby or child or children or childhood or infancy or infant or infants or juvenile or juveniles or kid or kids or newborn or new-born or newborns or new-borns or paediatric or paediatrics or pediatric or pediatrics or picu or perinatal or preadolescen* or pubescence or pubescent or school child or schoolchild or schoolchildren or teen or teens or teenager or teenagers or toddler or toddlers or youth or youths).mp. |
| 11 | 9 and 10 |
| 12 | limit 11 to english language |

CINAHL Complete (EBSCOhost)

| S1 | TI (long N3 (covid* or ncov* or novel coronavirus or novel betacoronavirus or sars-ncov-2 or sars-cov-2)) OR AB (long N3 (covid* or ncov* or novel coronavirus or novel betacoronavirus or sars-ncov-2 or sars-cov-2)) |
| --- | --- |
| S2 | TI (persist* N3 (covid* or ncov* or novel coronavirus or novel betacoronavirus or sars-ncov-2 or sars-cov-2)) OR AB (persist* N3 (covid* or ncov* or novel coronavirus or novel betacoronavirus or sars-ncov-2 or sars-cov-2)) |
| S3 | TI (chronic N3 (covid* or ncov* or novel coronavirus or novel betacoronavirus or sars-ncov-2 or sars-cov-2)) OR AB (chronic N3 (covid* or ncov* or novel coronavirus or novel betacoronavirus or sars-ncov-2 or sars-cov-2)) |
| S4 | TI ((long term or long-term or longterm) N3 effect* N3 (covid* or ncov* or novel coronavirus or novel betacoronavirus or sars-ncov-2 or sars-cov-2)) OR AB ((long term or long-term or longterm) N3 effect* N3 (covid* or ncov* or novel coronavirus or novel betacoronavirus or sars-ncov-2 or sars-cov-2)) |
| S5 | TI (sequela* N3 (covid* or ncov* or novel coronavirus or novel betacoronavirus or sars-ncov-2 or sars-cov-2)) OR AB (sequela* N3 (covid* or ncov* or novel coronavirus or novel betacoronavirus or sars-ncov-2 or sars-cov-2)) |
| S6 | TI ((post acute or post-acute or postacute) N3 (covid* or ncov* or novel coronavirus or novel betacoronavirus or sars-ncov-2 or sars-cov-2)) OR AB ((post acute or post-acute or postacute) N3 (covid* or ncov* or novel coronavirus or novel betacoronavirus or sars-ncov-2 or sars-cov-2)) |
| S7 | TI ((longhaul* or long haul* or long-haul*) N3 (covid* or ncov* or novel coronavirus or novel betacoronavirus or sars-ncov-2 or sars-cov-2)) OR AB ((longhaul* or long haul* or long-haul*) N3 (covid* or ncov* or novel coronavirus or novel betacoronavirus or sars-ncov-2 or sars-cov-2)) |
| S8 | TI ((post-covid* or postcovid*) N2 (syndrome or condition)) OR AB ((post-covid* or postcovid*) N2 (syndrome or condition)) |
| S9 | S1 OR S2 OR S3 OR S4 OR S5 OR S6 OR S7 OR S8 |
| S10 | MH (pediatrics+ or child+ or adolescence+ or infant+) |
| S11 | TI (adolescence or adolescent or adolescents or babies or baby or child or children or childhood or infancy or infant or infants or juvenile or juveniles or kid or kids or newborn or new-born or newborns or new-borns or paediatric or paediatrics or pediatric or pediatrics or picu or perinatal or preadolescen* or pubescence or pubescent or school child or schoolchild or schoolchildren or teen or teens or teenager or teenagers or toddler or toddlers or youth or youths) OR AB (adolescence or adolescent or adolescents or babies or baby or child or children or childhood or infancy or infant or infants or juvenile or juveniles or kid or kids or newborn or new-born or newborns or new-borns or paediatric or paediatrics or pediatric or pediatrics or picu or perinatal or preadolescen* or pubescence or pubescent or school child or schoolchild or schoolchildren or teen or teens or teenager or teenagers or toddler or toddlers or youth or youths) |
| S12 | S10 OR S11 |
| S13 | S9 AND S12 |
| S14 | Limiters – English language |

Web of Science Core Collection (Clarivate)

| 1 | TS = (long NEAR/3 (covid* or ncov* or “novel coronavirus” or “novel betacoronavirus” or sars-ncov-2 or sars-cov-2)) |
| --- | --- |
| 2 | TS = (persist* NEAR/3 (covid* or ncov* or “novel coronavirus” or “novel betacoronavirus” or sars-ncov-2 or sars-cov-2)) |
| 3 | TS = (chronic NEAR/3 (covid* or ncov* or “novel coronavirus” or “novel betacoronavirus” or sars-ncov-2 or sars-cov-2)) |
| 4 | TS= ((“long term” or long-term or longterm) NEAR/3 effect* NEAR/3 (covid* or ncov* or “novel coronavirus” or “novel betacoronavirus” or sars-ncov-2 or sars-cov-2)) |
| 5 | TS = (sequela* NEAR/3 (covid* or ncov* or “novel coronavirus” or “novel betacoronavirus” or sars-ncov-2 or sars-cov-2)) |
| 6 | TS = ((“post acute” or post-acute or postacute) NEAR/3 (covid* or ncov* or “novel coronavirus” or “novel betacoronavirus” or sars-ncov-2 or sars-cov-2)) |
| 7 | TS = ((longhaul* or “long haul” or long-haul*) NEAR/3 (covid* or ncov* or “novel coronavirus” or “novel betacoronavirus” or sars-ncov-2 or sars-cov-2)) |
| 8 | TS = ((post-covid* or postcovid*) NEAR/2 (syndrome or condition)) |
| 9 | #8 OR #7 OR #6 OR #5 OR #4 OR #3 OR #2 OR #1 |
| 10 | TS = (adolescence or adolescent or adolescents or babies or baby or child or children or childhood or infancy or infant or infants or juvenile or juveniles or kid or kids or newborn or new-born or newborns or new-borns or paediatric or paediatrics or pediatric or pediatrics or picu or perinatal or preadolescen* or pubescence or pubescent or “school child” or schoolchild or schoolchildren or teen or teens or teenager or teenagers or toddler or toddlers or youth or youths) |
| 11 | #10 AND #9 |
|  | English |

Cochrane Database of Systematic Reviews | Cochrane Central Register of Controlled Trials

| #1 | (long NEAR/3 (covid* or ncov* or “novel coronavirus” or “novel betacoronavirus” or sars-ncov-2 or sars-cov-2)):ti,ab,kw |
| --- | --- |
| #2 | (persist* NEAR/3 (covid* or ncov* or “novel coronavirus” or “novel betacoronavirus” or sars-ncov-2 or sars-cov-2)):ti,ab,kw |
| #3 | (chronic NEAR/3 (covid* or ncov* or “novel coronavirus” or “novel betacoronavirus” or sars-ncov-2 or sars-cov-2)):ti,ab,kw |
| #4 | ((“long term” or long-term or longterm) NEAR/3 effect* NEAR/3 (covid* or ncov* or “novel coronavirus” or “novel betacoronavirus” or sars-ncov-2 or sars-cov-2)):ti,ab,kw |
| #5 | (sequela* NEAR/3 (covid* or ncov* or “novel coronavirus” or “novel betacoronavirus” or sars-ncov-2 or sars-cov-2)):ti,ab,kw |
| #6 | ((“post acute” or post-acute or postacute) NEAR/3 (covid* or ncov* or “novel coronavirus” or “novel betacoronavirus” or sars-ncov-2 or sars-cov-2)):ti,ab,kw |
| #7 | ((longhaul* or “long haul” or long-haul*) NEAR/3 (covid* or ncov* or “novel coronavirus” or “novel betacoronavirus” or sars-ncov-2 or sars-cov-2)):ti,ab,kw |
| #8 | ((post-covid* or postcovid*) NEAR/2 (syndrome or condition)):ti,ab,kw |
| #9 | #8 OR #7 OR #6 OR #5 OR #4 OR #3 OR #2 OR #1 |
| #10 | (adolescence or adolescent or adolescents or babies or baby or child or children or childhood or infancy or infant or infants or juvenile or juveniles or kid or kids or newborn or new-born or newborns or new-borns or paediatric or paediatrics or pediatric or pediatrics or picu or perinatal or preadolescen* or pubescence or pubescent or “school child” or schoolchild or schoolchildren or teen or teens or teenager or teenagers or toddler or toddlers or youth or youths):ti,ab,kw |
| #11 | #10 AND #9 |

Appendix 2:

Case Definitions for Post-Acute Sequelae of COVID-19

| World Health Organization | The continuation or development of new symptoms 3 months after the initial SARS-CoV-2 infection, with these symptoms lasting for at least 2 months with no other explanation (51). |
| --- | --- |
| Center for Disease Control | Signs, symptoms and conditions that continue or develop at least four weeks after COVID-19 infection (52). |

Appendix 3: QUIPS risk of bias assessments for the included cross sectional and cohort studies (n = 15)

| **Study** | **Study Participation** | **Study Attrition** | **Prognostic Factor Measurement** | **Outcome Measurement** | **Study Confounding** | **Statistical Analysis & Reporting** | **Overall** |
| --- | --- | --- | --- | --- | --- | --- | --- |
| Fashina et al 2022 | Moderate | Moderate | Low | Moderate | High | Low | High |
| Enner et al 2022 | Moderate | Low | Low | Moderate | Low | Moderate | Moderate |
| Kahn et al 2022 | Moderate | High | Moderate | High | High | High | High |
| Buonsenso 2022 | Moderate | Moderate | Moderate | Low | Moderate | Moderate | High |
| Palacios et al 2022 | High | High | Moderate | Moderate | Moderate | Moderate | High |
| Morrow et al 2022 | High | High | High | Moderate | High | High | High |
| Bhargava et al 2022 | High | High | High | High | High | High | High |
| Maddux et al 2022 | Moderate | High | Low | Low | High | High | High |
| Hazan et al 2022 | Moderate | Low | Moderate | High | Low | Low | High |
| Messiah et al 2022 | Moderate | Low | High | Low | High | High | High |
| Drogalis-Kim et al 2022 | High | High | High | High | High | High | High |
| Ebell et al 2022 | Moderate | High | High | Moderate | Moderate | Moderate | High |
| Walsh-Messinger et al 2022 | Moderate | High | High | Moderate | Moderate | Moderate | High |
| Rao et al 2022 | Moderate | Low | Low | Moderate | Moderate | Low | Moderate |
| Chou et al 2022 | Moderate | High | Low | Low | Moderate | Low | High |
| LeftinDubkin et al 2022 | Moderate | High | High | High | High | High | High |

Appendix 4: Study characteristics table

| **Study ID** | **Overall Bias Assessment Score** | **Number of patients with PASC** | **Aim of study** | **Study design** | **PASC Symptoms Experienced** | **Duration of PASC symptoms** | | **Diagnostics** | **Interventions** | **Past Medical History** | **Patient Acute COVID symptoms** |
| --- | --- | --- | --- | --- | --- | --- | --- | --- | --- | --- | --- |
| Younger 2022 | N/A | 1 | Case study of teen with PASC manifesting as progressive post-infectious central, peripheral, and ANS sxs | Case report | Progressive burning pain, limb weakness, slurred speech, impaired cognition, stocking sensory loss to vibration, cold temperature and pin prick, areflexia; delium | > 15 months | | EDX showed mixed chronic distal demyelinating and axonal changes; blood pressure and heart rate diagnostic of orthostatic intolerance; PET/MRI showed hypometabolism of bilateral anterior and mesial temporal, superior parietal, and lateral occipital lobes,  anterior cingulate cortices, and the cerebellar hemispheres (Volumetric analysis suggested left hippocampal and temporal hippocampal volumes to be<5% of age-matched  controls); Mayo Clinic ENS2 panel showed a serum glutamic acid dehydrogenase (GAD)65 antibody titer of 0.03 nmol/L (reference value0.02); Lumbar puncture showed a total protein of 136 mg/dL, otherwise normal. EEG showed no evidence of localized slowing or epileptiform activity. | From October 2020 to February 2021, she received 2 g/kg/month of intravenous immune globulin (IVIg) therapy with overall  initial clinical improvement | none | loss of taste, and excessive fatigue (march 2020 diagnosis) |
| Walsh-Messinger 2021 | High | 22 | Investigates the prevalence and features of post-COVID syndrome in a sample of university students with mild to moderate acute illness severity | Cross sectional study | impaired concentration (~60%), headaches (~60%), rhinitis (~50%), impaired sleep (~50%), dyspnea ~50%, brain fog ~50%, appetite loss, fatigue, chest pain, cough, myalgia, diarrhea, olfacotry impairment, LAD, gustatory impairment, constipation, fever, tachycardia, pharyngitis, rash, HTN , hypoxemia | more than 50 days later | None described | | N/A | 36% w/ allergic rhinitis; 1 person with HTN, 1w/ IBS, 1 w/ endometriosis and fibromyalgia; 3 with asthma | PASC group: 36% mild, 59% moderate, 4.5% severe; Recovered group: 76% mild; Mild symptoms included chest pain, fatigue, fever, olfactory impairment, fever, headaches, diarrhea, congestion, sore throat, gustatory impairment |
| Verma 2021 | N/A | N/A | Editorial about Long COVID in kids and potential effects it can have on children vs adults. | United States | Not described | N/A | N/A | | N/A | N/A |  |
| Thallapureddy 2022 | N/A | 5 (described in Lindan et al 2021) | Systematic review of pediatric Long COVID | United States-- among other countries | Encephalopathy, gait impairment, headache, neck pain, cerebellar ataxia, muscle weakness, stupor, pyramidal signs, visual hallucinations, meningismus, AMS, 4 limb motor/bladder dysfunction, ophthalmoplegia, flaccid paralysis, ptosis, hypotonia, seizures | N/A | MRI | | N/A | N/A | 19.2% asymptomatic; fever, and upper respiratory symptoms with/without gastro-intestinal symptoms, sore throat, shortness of breath, headache |
| Simmons 2022 | N/A | 3 | Highlight the importance of awareness of the neurological sequelae of novel coronavirus infection in children and adolescents. | Case series | Headaches—both bifrontal and bitemporal with without radiation and/or pounding; neck tightness; photophobia; daily headaches; dizziness; blurred vision; appetite loss | -7+months -8 months-5-14 monts | Not described | | Non-pharmacologic: sleep hygiene, adequate water intake, limiting screen time, limiting caffeine; Acupuncture, chiropractic medicine, and massage therapy were ineffective in improving symptoms. Pharmacologic: Combination of acetaminophen, aspirin, and caffeine or ibuprofen, Triptans for migraine rescue; Verapamil tried and ineffective in 1 patient. | Depression; dull constant frontal headache of 4/10 severity after a concussion with no associated episodic syndromes, which lasted approximately six months (this was 1 yr before COVID) | Seven days of headache, extremity numbness, fatigue, and neck pain, congestion, anosmia, rhinorrhea, appetite loss |
| Roesler 2022 | N/A | N/A | Editoiral describing how school nurses should be involved with Long COVID in children | Text and opinion | N/A | N/A | Not described | | None | None | N/A |
| Rao 2021 (preprint) | Moderate | N/A | What are the incidence and clinical features of post-acute sequelae of SARS-CoV-2 infection (PASC) in children? | Cohort study | Change of smell or taste, loss of smell, hair loss, chest pain, abnormal LFTs, fatigue, malaise, fever and chills, cardio respiratory symptoms, diarrhea, myocarditis, ARDS, disorders of teeth/gingiva, other/ill defined heart disease, fluid and electrolyte disturbances , anxiety, headaches, vertigo paresthesias, memory loss and brain fog, shortness of breath | 28-179 days | N/A | | N/A | obesity | most with mild illness, some with hospitalization (~1-2% admitted to ICU) |
| Rao 2022 | Moderate | N/A | What are the incidence and clinical features of post-acute sequelae of SARS-CoV-2 infection (PASC) in children? | Cohort study | Change of smell or taste, loss of smell, hair loss, chest pain, abnormal LFTs, fatigue, malaise, fever and chills, cardio respiratory symptoms, diarrhea, myocarditis, ARDS, disorders of teeth/gingiva, other/ill defined heart disease, fluid and electrolyte disturbances , anxiety, headaches, vertigo paresthesias, memory loss and brain fog, shortness of breath | 28-179 days | N/A | | N/A | Comorbid complex disease | most with mild illness, some with hospitalization (~1-2% admitted to ICU) |
| Palacios 2022 | High | 66 | To describe long‚ term subjective and objective pulmonary abnormalities after SARS-CoV-2 infection in pediatric populations | Cohort study | Chest pain (61%), cough (30.5%), dyspnea (51.2%), sob during exercise (90.2%) | N/A | 6-minute walk test with Borg rating of perceived exertion, chest x-ray, pre and postbronchodilator spirometry, plethysmography, and diffusion capacity of lung for carbon monoxide (DLCO) testing, laryngoscopy | | 43% of individuals were prescribed an inhaled corticosteroid or inhaled corticosteroid/long acting beta agonist combination and noted 30.8% w/ bronchodialator response; 13% of individuals were diagnosed with paradoxical vocalfold motion disorder (PVFMD) in conjunction with ENT and were treated with speech therapy | 29%(n = 24)asthma, with the majority (75.0%, n = 18) characterized as 75% mild intermittent, 20.8% mild persistent, 4.2% moderate persistent; 20.7% obese; 27% anxiety; 80.5% competitive athlete | most common: cough and shortness of breath; fever 50; anosmia 47.6%; upper respiraotyr infection 58%; headache 52% |
| Nevid 2021 | N/A | 3 | Describes three cases of adolescents who had dyspnea noted months after COVID-19 infection | Case series | dyspnea on exertion; case 3 w/ dyspnea at rest and exertion | "months" | Impulse oscillometry, exercise laryngoscopy, pulmonary function testing | | budesonide-formoterol twice daily and albuterol prior to exercise with significant clinical improvement; fluticasone-propionate and salmeterol twice daily | N/A | N/A |
| Morrow 2021 | High | 8 | To describe a cohort of patients with PASC as well as the development of this institution's PASC clinic | Cohort study | Fatigue, muscle weakness, dizziness, headaches, brain fog, non-epileptic seizures (in pt without seizure disorder), panic attacks, abnormal movements, trouble breathing, pain, deconditioning, abdominal pain, facial flushing, rashes/hives, sore throat, swollen glands, diarrhea, chest pain, school concerns, cognitive difficulties, dizziness/lightheadedness, chest pain/pressure/tightness, palpitations | N/A | 10 min standing tolerance test, PedsQL (for quality of life assessment), electrocardiogram, chest radiograph, echocardiogram, cardiac enzymes, Holter monitor, cardiac stress test, pulmonary function test, and peak flow meter, neuropsychiatric testing | | Physical therapy, social worker meeting for coping skills and support, psychologist assessment and intervention (ie brief CBT, or other therapeutic techniques), neuropsychiatric assessment and possible intervention, bronchodialator helped 1 pt w/ persistent pulmonary symptoms, graded exercise therapy, sleep hygiene, CBT for fatigue | Type 1 diabetes, speech delay, anxiety, asthma, seasonal allergies, dyslexia, Eosinophilic esophagitis, vitilito, sensory processing disorder, malabsorpiton, scoliosis, migraine, concussions, sensory integration disorder, ADHD/ADD concerns prior to COVID; 6/8 with mood and/or anxiety concerns prior to covid, 3/8 with neurodevelopmental concerns | 1 pt hospitalized for MISC; rest with mild initial illness |
| Malone 2022 | N/A | N/A | Develop consensus guidelines for assessment and management of PASC in Children and adolescents. | Consensus guideline | N/A | N/A | Extensive and thorough description of work-up going through each organ system. | | Interventions based on organ system involved. | N/A | N/A |
| Maddux 2022 | High | 80 (32 from acute COVID, 48 from MISC) | To characterize in the US children hospitalized with acute severe COVID-19 or MIS-C 2 or more months after hospital admission, and identify factors associated with ongoing symptoms or activity impairment | Cohort study | Fatigue or weakness, shortness of breath, cough, headache, myalgia, fever, loss of smell, diarrhea, sore throat, loss of taste, vomiting | about 2 months | N/A | | not explicititely described although speculate that vaccination may help since vaccinated children had less severe acute illness | 39% obsese in acute COVID, 50% obese MISC; asthma in MISC | All patients in this study were hospitalized; Patients with persistent MISC symptoms had longer initial hospital stays |
| LeftinDobkin 2021 (poster abstract and then full paper from Pediatric Pulmonology) | High | 29 | to describe baseline health data and respiratory findings in a cohort of pediatric patients experiencing prolonged symptoms following acute COVID-19 infection | Cohort study | 96.6% w/ persistent dyspnea an/or exertional dyspnea, 51.7% with chronic cough, 48.3% w/ exercise intolerance, 13.8% w fatigue, 1 pt with ongoing oxygen requirement | N/A | spirometry, plethysmography, diffusion studies, and six- walk tests (6MWT), pulse oximetry | | improvement following bronchodilator administration was observed in 38.1% of the 21 subjects who underwent post bronchodilator testing ( four of whom had known underlying asthma and four of whom did not) | Overweight (24.1%; body mass index [BMI] between 85th and 95th percentile) or obese (37.9%; BMI >95th percentile). Baseline atopy, defined as presence of asthma, eczema, and/or allergic rhinitis, was present in 65.5% of subjects. | fever (69%), cough (55.2%), dyspnea (48.3%), ageusia/anosmia (41.4%) and myalgia (37.9%);four patients (13.8%) required hospitalization during initial illness; 1 additional pt with MIS-C; duration of illness was 13.4 +/- 11 days |
| Khan 2022 | Moderate | 3 | To study the incidence, demographics, clinical characteristics, management, and outcomes of pediatric SARS-CoV-2-associated diagnoses. | Cohort study | myalgia, fatigue, and brain fog | 6 weeks at least | N/A | | patients were treated symptomatically with academic accommodations at school | N/A | 67% symptomatic-fever, skin rash, URI sxs, GI sxs, LRI, arthralgias, extremity swelling, conjunctivitis, encephalopathy, oral mucosa involvement |
| Hanson 2022 | N/A | N/A | What are the extent and nature of the most common long COVID symptoms by country in   2020 and 2021? | meta-analysis and systematic review | fatigue, cognitive problems, shortness of breath, memory problems, fatigue, dyspnea s | ranged up to 12 months | N/A | | N/A | N/A | mild illness |
| Hazan 2022 | High | 10 | The aim of this study is to identify markers for severe acute disease and chronic long-term complications of COVID-19 in a hospitalized pediatric population | Cohort study | 12% of the symptomatic patients had chronic respiratory complications (persistent tachypnea, persistent hypoxemia, and/or chronic ground glass opacities in chest CT); non-respiratory symptoms (13%): recurrent leg pain w/ erythema, Guillain Barre, adrenal insufficiency, MIS-C | N/A | symptomatic patients who had radiologic follow up, 16.7% were found to have chronic chest radiograph abnormalities (chronic atelectesis, chronic opacities c/w scarring) | | Oxygen for hypoxemic patients | pulmonary co-morbidity (associated with increased risk for chronic disease), obesity | All patients in this article were hospitalized; 66% symptomatic COVID (rest were there for things like surgery or trauma); 31% were admitted to PICU |
| Haelle 2021 | N/A | N/A | Describe prevalence of Long COVID in children | Description of a study | Respiratory symptoms in 2% of cases, systemic (such as fatigue and fever) in 2% of cases, neurologic (such as headache, seizures, and continued loss of taste or smell) in 1% of cases, and psychological (such as new-onset depression and anxiety) in 1% of cases; children who presented to the ED with at least seven symptoms were four times more likely to have long-term symptoms than those who presented with fewer symptoms (OR, 4.02; P = .01) | N/A | N/A | | N/A | N/A | both severe and asymptomatic were included |
| Gupta 2022 | N/A | 373 w/ neuro involvement; 1 w. memory dificulties and agitation; 306 w/ difficulty concentrationg, 234 w/ memory problems, 204 w/ every day task challenges, 410 with fatigue | To review the empirical evidence regarding neuropsychaitric illness (Long COVID) in children and adolescents post-severe acute respiratory coronavirus infection | Systematic review | 33% of pts in US based study with neurologic or psychiatric symptoms (including: OCD symptoms, emotional lability, facial motor tics, guttural tics, aggressiveness, irritability, inattentiveness, inappetence and lack of attention); prolonged HAs, difficulty concentrating, brain fog, memory loss; GBS, encephalopathy, demyelination of CNS, stroke, and acute fulminant edema | 6 months for some | N/A | | N/A | N/A | some with severe COVID-19 disease |
| Fashina 2022 **(**poster abstract extracted from COVIDENCE, J. PIDS paper 2023 publication also added) | High | 37 | Explore the clinical spectrum and out-comes of children and adolescents diagnosed with PASC within a large healthcare system | Cross sectional study | fatigue, dyspnea, HAs, anosmia, chest pain, dizziness/lightheadedness, brain fog, sleep disturbance, loss of appetite, recurrent fever, abd pain, palpitations, myalgias | N/A | N/A | | N/A | atopy (32%), asthma (22%), obesity (12%); 28% w/ no past medical history | 88% w/ mild illness, 12% w/hospitalization |
| Ebell 2022 | High | 14 | To determine the prevalence of previous infection, risk factors for infection, and the prevalence of persistent symptoms following infection among university students | Cross sectional study | fatigue, exertional dyspnea, MSK pain, brain fog, anxiety | surveyed patinets up to 2 months | N/A | | N/A | N/A | fever, cough, dyspnea, fatigue |
| Drogalis-Kim 2022 | High | 9 | To highlight the phenomenon of dysautonomia after acute SARS-CoV-2 infection and its response to therapy | Case series | daily dizziness, lightheadedness, unsteadiness with positional changes, fatigue, headaches, difficulty concentrating, dyspnea on exertion, nausea/vomiting/depressed appetite, anxiety |  | Orthostatic hypotension testing (Active Standing Test), EKG (all normal), cardiac monitor, cardio-pulm exercise testing, +/- laboratory testing | | Non-pharmacologic therapy: 1. 2.5-3L of water per day 2. 8-10g sodium per day 3. compression garments 4. improved sleep hygiene 5. daily aerobic exercise regimen (can start with recumbent or semi-recumbent if extremely symptomatic 6. lower extremity strengthening exercises; Pharmacologic therapy: for POTS: fludrocortisone, atenolol, midodrine; Other: CBT for orthostatic intolerance , acupuncture | 3 pts w with past medical history of anxiety; 1 with occasional dizziness prior to COVID, 1 with asthma | All experienced mild acute symptoms; 6 w/ fever, 5 with URI sxs, 1 w/ GI sxs, 5 w/ anosmia or anguesia |
| Brackel 2022 | N/A | 431 | To assess the currently available pediatric international long-COVID care programs and explore the characteristics of their patient cohorts | Cohort study | fatigue, headaches, concentration difficulties, dyspnea and sleep disturbances. 5-37% of patients had severe limitations in daily life | some patients suffered from complaints for more than twelve weeks | N/A | | N/A | most commonly atopic syndrome, asthma and prematurity | Most patients (90-100%) suffered from asymptomatic or mild acute COVID-19 |
| Bhargava 2021 | High | 64 | Characterize symptoms onset, symptoms, and duration of symptoms in pediatric PASC from NJ clinic | Cohort study | 55.6% experienced fatigue, 50% experienced shortness of breath or cough, 50% experienced brain fog, 33% chest pain and 44.4% experienced anxiety and/or depression | at least 4 weeks | N/A | | N/A | N/A | N/A |
| Barmherzig 2021 | N/A | 17 | Describe headaches in pediatric patients following COVID-19 | Case series | Moderate to severe daily headaches(frontal or holocephalic- 94% (16/17), fatigue, decreased exercise tolerance, myalgias, cognitive complaints, dizziness, altered smell, emotional changes, poor sleep, and GI symptoms | up to 6 months | Neuroimaging following infection (n = 4), all had normal studies or chronic findings unrelated to COVID-19. | | All patients tried anti-inflammatory drugs (NSAIDs) without benefit. Other treatments offered following initial presentation included oral steroids (n = 3), peripheral nerve blocks (n = 3), and Emergency Room or inpatient migraine treatments such as intravenous ketorolac,metoclopramide and methylprednisolone (n = 5). Nerve blocks were not associated with meaningful benefit in all three patients who received this intervention. Nutraceutical supplements most commonly recommended were magnesium, vitamin D, and melatonin. Preemptive treatment with zonisamide and gabapentin were associated with a more favorable response (n = 7). | Background history of episodic headaches preceding COVID infection was present in 83.3% (15/17) of included patients | Headache |
| Chou 2022 | High | N/A | Assess whether SARS-CoV-2 is an asthma trigger resulting in poor asthma control | Cross sectional study | asthma symptoms | N/A | N/A | | N/A | Asthma | N/A |
| Rao 2024 | N/A | N/A | To identify diagnosed symptoms, health conditions and medications in children with PASC. | Cohort study | loss of taste or smell, myocarditis, chest pain, hair loss, abnormal liver enzymes, skin rashes, fatigue, maliase, fever and chills, GI issues, disorders of teeth or gingiva, anxiety | N/A | N/A | | N/A | N/A | N/A |
| Enner 2022 | Moderate | 47-- all with MISC related PASC | describe long term sequelae of MISC | Cohort study | neurologic symptoms, psychiatric symptoms, sleep disturbances, | N/A | N/A | | N/A | N/A | all hospitalized for MIS-C |
| Messiah 2022 | High | 111 (11 from MISC Cohort and 110 from non-MISC Cohort) | Comparison of Long-Term Complications of COVID-19 Illness among a Diverse Sample of Children by MIS-C Status | Cross sectional study | Neurologic symptoms, seizure, headaches, attention difficulty, sleep symptoms, mood/ behavioral changes | N/A | N/A | | N/A | N/A | 84% of patients were hospitalized with acute COVID-19 illness |
|  |  |  |  |  |  |  |  | |  |  |  |
| Per Reviewers-- |  |  |  |  |  |  |  | |  |  |  |
| Delagu et al 2024 |  | 56 | Describe autonomic dysfunction in pediatric PASC patients | case control | pediatric patients with long COVID had significant changes in HRV variables compared to healthy controls: significantly lower r-MSSD (root mean square of successive RR interval differences, 47.4 ± 16.9 versus 60.4 ± 29.1, *p* = 0.02), significant higher values VLF (very low frequency, 2077.8 ± 1023.3 versus 494.3 ± 1015.5 ms, *p* = 0.000), LF (low frequency, 1340.3 ± 635.6 versus 354.6 ± 816.8 ms, *p* = 0.000), and HF (high frequency, 895.7 ± 575.8 versus 278.9 ± 616.7 ms, *p* = 0.000). No significant differences were observed between the two groups both in systolic and diastolic parameters by echocardiography--> relative abundance of parasympathetic tone |  | important because it describes Holter EKG monitoring and echos done to all pedi patients in their cohort. Found abnormalities | |  |  |  |
| Baldi et al 2024 |  | 61 | Describes cardiopulomonary exercise testing in pedi PASC patients | case control | Children with LC have a reduced VO2 peak (Oxygen uptake at peak of exercise), abnormal cardiovascular efficiency (VO2/HR% pred), pathological VE/VCO slope (indicative of the possible presence of ventilatory inefficiency/pulmonary vascular commitment if higher than 30), and abnormally reduced slope of VO2 work (indicative of muscle deconditioning). |  | LC patients who presented mostly cardiorespiratory symptoms had a corresponding pattern of functional limitation at CPET characterized by signs of cardiogenic inefficiency and possible pulmonary vascular involvement; on the other hand, those who mostly presented asthenia (100%) and musculoskeletal symptoms (86%) presented a corresponding CPET pattern suggestive of signs of muscle deconditioning as well as an early anaerobic threshold | |  |  |  |
| Buonsenso et al 2022 |  | 51 |  |  | some children with Long COVID show an abnormal switch from innate to adaptive immune responses, documented by the low representation of effector T cells and of IgD- B cells. |  |  | |  |  |  |

| **Study ID** | **Overall Bias Assessment Score** | **Number of patients with PASC** | **Aim of study** | **Study design** | **PASC Symptoms Experienced** | **Duration of PASC symptoms** | **Diagnostics** | **Interventions** | **Past Medical History** | **Patient Acute COVID symptoms** |
| --- | --- | --- | --- | --- | --- | --- | --- | --- | --- | --- |
| Younger 2022 | N/A | 1 | Case study of teen with PASC manifesting as progressive post-infectious central, peripheral, and ANS sxs | Case report | Progressive burning pain, limb weakness, slurred speech, impaired cognition, stocking sensory loss to vibration, cold temperature and pin prick, areflexia; delium | > 15 months | EDX showed mixed chronic distal demyelinating and axonal changes; blood pressure and heart rate diagnostic of orthostatic intolerance; PET/MRI showed hypometabolism of bilateral anterior and mesial temporal, superior parietal, and lateral occipital lobes,  anterior cingulate cortices, and the cerebellar hemispheres (Volumetric analysis suggested left hippocampal and temporal hippocampal volumes to be<5% of age-matched  controls); Mayo Clinic ENS2 panel showed a serum glutamic acid dehydrogenase (GAD)65 antibody titer of 0.03 nmol/L (reference value0.02); Lumbar puncture showed a total protein of 136 mg/dL, otherwise normal. EEG showed no evidence of localized slowing or epileptiform activity. | From October 2020 to February 2021, she received 2 g/kg/month of intravenous immune globulin (IVIg) therapy with overall  initial clinical improvement | none | loss of taste, and excessive fatigue (march 2020 diagnosis) |
| Walsh-Messinger 2021 | High | 22 | Investigates the prevalence and features of post-COVID syndrome in a sample of university students with mild to moderate acute illness severity | Cross sectional study | impaired concentration (~60%), headaches (~60%), rhinitis (~50%), impaired sleep (~50%), dyspnea ~50%, brain fog ~50%, appetite loss, fatigue, chest pain, cough, myalgia, diarrhea, olfacotry impairment, LAD, gustatory impairment, constipation, fever, tachycardia, pharyngitis, rash, HTN , hypoxemia | more than 50 days later | None described | N/A | 36% w/ allergic rhinitis; 1 person with HTN, 1w/ IBS, 1 w/ endometriosis and fibromyalgia; 3 with asthma | PASC group: 36% mild, 59% moderate, 4.5% severe; Recovered group: 76% mild; Mild symptoms included chest pain, fatigue, fever, olfactory impairment, fever, headaches, diarrhea, congestion, sore throat, gustatory impairment |
| Verma 2021 | N/A | N/A | Editorial about Long COVID in kids and potential effects it can have on children vs adults. | United States | Not described | N/A | N/A | N/A | N/A |  |
| Thallapureddy 2022 | N/A | 5 (described in Lindan et al 2021) | Systematic review of pediatric Long COVID | United States-- among other countries | Encephalopathy, gait impairment, headache, neck pain, cerebellar ataxia, muscle weakness, stupor, pyramidal signs, visual hallucinations, meningismus, AMS, 4 limb motor/bladder dysfunction, ophthalmoplegia, flaccid paralysis, ptosis, hypotonia, seizures | N/A | MRI | N/A | N/A | 19.2% asymptomatic; fever, and upper respiratory symptoms with/without gastro-intestinal symptoms, sore throat, shortness of breath, headache |
| Simmons 2022 | N/A | 3 | Highlight the importance of awareness of the neurological sequelae of novel coronavirus infection in children and adolescents. | Case series | Headaches—both bifrontal and bitemporal with without radiation and/or pounding; neck tightness; photophobia; daily headaches; dizziness; blurred vision; appetite loss | -7+months  -8 months -5-14 monts | Not described | Non-pharmacologic: sleep hygiene, adequate water intake, limiting screen time, limiting caffeine; Acupuncture, chiropractic medicine, and massage therapy were ineffective in improving symptoms. Pharmacologic: Combination of acetaminophen, aspirin, and caffeine or ibuprofen, Triptans for migraine rescue; Verapamil tried and ineffective in 1 patient. | Depression; dull constant frontal headache of 4/10 severity after a concussion with no associated episodic syndromes, which lasted approximately six months (this was 1 yr before COVID) | Seven days of headache, extremity numbness, fatigue, and neck pain, congestion, anosmia, rhinorrhea, appetite loss |
| Roesler 2022 | N/A | N/A | Editoiral describing how school nurses should be involved with Long COVID in children | Text and opinion | N/A | N/A | Not described | None | None | N/A |
| Rao 2021 (preprint) | Moderate | N/A | What are the incidence and clinical features of post-acute sequelae of SARS-CoV-2 infection (PASC) in children? | Cohort study | Change of smell or taste, loss of smell, hair loss, chest pain, abnormal LFTs, fatigue, malaise, fever and chills, cardio respiratory symptoms, diarrhea, myocarditis, ARDS, disorders of teeth/gingiva, other/ill defined heart disease, fluid and electrolyte disturbances , anxiety, headaches, vertigo paresthesias, memory loss and brain fog, shortness of breath | 28-179 days | N/A | N/A | obesity | most with mild illness, some with hospitalization (~1-2% admitted to ICU) |
| Rao 2022 | Moderate | N/A | What are the incidence and clinical features of post-acute sequelae of SARS-CoV-2 infection (PASC) in children? | Cohort study | Change of smell or taste, loss of smell, hair loss, chest pain, abnormal LFTs, fatigue, malaise, fever and chills, cardio respiratory symptoms, diarrhea, myocarditis, ARDS, disorders of teeth/gingiva, other/ill defined heart disease, fluid and electrolyte disturbances , anxiety, headaches, vertigo paresthesias, memory loss and brain fog, shortness of breath | 28-179 days | N/A | N/A | Comorbid complex disease | most with mild illness, some with hospitalization (~1-2% admitted to ICU) |
| Palacios 2022 | High | 66 | To describe long‚ term subjective and objective pulmonary abnormalities after SARS-CoV-2 infection in pediatric populations | Cohort study | Chest pain (61%), cough (30.5%), dyspnea (51.2%), sob during exercise (90.2%) | N/A | 6-minute walk test with Borg rating of perceived exertion, chest x-ray, pre and postbronchodilator spirometry, plethysmography, and diffusion capacity of lung for carbon monoxide (DLCO) testing, laryngoscopy | 43% of individuals were prescribed an inhaled corticosteroid or inhaled corticosteroid/long acting beta agonist combination and noted 30.8% w/ bronchodialator response; 13% of individuals were diagnosed with paradoxical vocalfold motion disorder (PVFMD) in conjunction with ENT and were treated with speech therapy | 29%(n = 24)asthma, with the majority (75.0%, n = 18) characterized as 75% mild intermittent, 20.8% mild persistent, 4.2% moderate persistent; 20.7% obese; 27% anxiety; 80.5% competitive athlete | most common: cough and shortness of breath; fever 50; anosmia 47.6%; upper respiraotyr infection 58%; headache 52% |
| Nevid 2021 | N/A | 3 | Describes three cases of adolescents who had dyspnea noted months after COVID-19 infection | Case series | dyspnea on exertion; case 3 w/ dyspnea at rest and exertion | "months" | Impulse oscillometry, exercise laryngoscopy, pulmonary function testing | budesonide-formoterol twice daily and albuterol prior to exercise with significant clinical improvement; fluticasone-propionate and salmeterol twice daily | N/A | N/A |
| Morrow 2021 | High | 8 | To describe a cohort of patients with PASC as well as the development of this institution's PASC clinic | Cohort study | Fatigue, muscle weakness, dizziness, headaches, brain fog, non-epileptic seizures (in pt without seizure disorder), panic attacks, abnormal movements, trouble breathing, pain, deconditioning, abdominal pain, facial flushing, rashes/hives, sore throat, swollen glands, diarrhea, chest pain, school concerns, cognitive difficulties, dizziness/lightheadedness, chest pain/pressure/tightness, palpitations | N/A | 10 min standing tolerance test, PedsQL (for quality of life assessment), electrocardiogram, chest radiograph, echocardiogram, cardiac enzymes, Holter monitor, cardiac stress test, pulmonary function test, and peak flow meter, neuropsychiatric testing | Physical therapy, social worker meeting for coping skills and support, psychologist assessment and intervention (ie brief CBT, or other therapeutic techniques), neuropsychiatric assessment and possible intervention, bronchodialator helped 1 pt w/ persistent pulmonary symptoms, graded exercise therapy, sleep hygiene, CBT for fatigue | Type 1 diabetes, speech delay, anxiety, asthma, seasonal allergies, dyslexia, Eosinophilic esophagitis, vitilito, sensory processing disorder, malabsorpiton, scoliosis, migraine, concussions, sensory integration disorder, ADHD/ADD concerns prior to COVID; 6/8 with mood and/or anxiety concerns prior to covid, 3/8 with neurodevelopmental concerns | 1 pt hospitalized for MISC; rest with mild initial illness |
| Malone 2022 | N/A | N/A | Develop consensus guidelines for assessment and management of PASC in Children and adolescents. | Consensus guideline | N/A | N/A | Extensive and thorough description of work-up going through each organ system. | Interventions based on organ system involved. | N/A | N/A |
| Maddux 2022 | High | 80 (32 from acute COVID, 48 from MISC) | To characterize in the US children hospitalized with acute severe COVID-19 or MIS-C 2 or more months after hospital admission, and identify factors associated with ongoing symptoms or activity impairment | Cohort study | Fatigue or weakness, shortness of breath, cough, headache, myalgia, fever, loss of smell, diarrhea, sore throat, loss of taste, vomiting | about 2 months | N/A | not explicititely described although speculate that vaccination may help since vaccinated children had less severe acute illness | 39% obsese in acute COVID, 50% obese MISC; asthma in MISC | All patients in this study were hospitalized; Patients with persistent MISC symptoms had longer initial hospital stays |
| LeftinDobkin 2021 (poster abstract and then full paper from Pediatric Pulmonology) | High | 29 | to describe baseline health data and respiratory findings in a cohort of pediatric patients experiencing prolonged symptoms following acute COVID-19 infection | Cohort study | 96.6% w/ persistent dyspnea an/or exertional dyspnea, 51.7% with chronic cough, 48.3% w/ exercise intolerance, 13.8% w fatigue, 1 pt with ongoing oxygen requirement | N/A | spirometry, plethysmography, diffusion studies, and six- walk tests (6MWT), pulse oximetry | improvement following bronchodilator administration was observed in 38.1% of the 21 subjects who underwent post bronchodilator testing ( four of whom had known underlying asthma and four of whom did not) | Overweight (24.1%; body mass index [BMI] between 85th and 95th percentile) or obese (37.9%; BMI >95th percentile). Baseline atopy, defined as presence of asthma, eczema, and/or allergic rhinitis, was present in 65.5% of subjects. | fever (69%), cough (55.2%), dyspnea (48.3%), ageusia/anosmia (41.4%) and myalgia (37.9%);four patients (13.8%) required hospitalization during initial illness; 1 additional pt with MIS-C; duration of illness was 13.4 +/- 11 days |
| Khan 2022 | Moderate | 3 | To study the incidence, demographics, clinical characteristics, management, and outcomes of pediatric SARS-CoV-2-associated diagnoses. | Cohort study | myalgia, fatigue, and brain fog | 6 weeks at least | N/A | patients were treated symptomatically with academic accommodations at school | N/A | 67% symptomatic -fever, skin rash, URI sxs, GI sxs, LRI, arthralgias, extremity swelling, conjunctivitis, encephalopathy, oral mucosa involvement |
| Hanson 2022 | N/A | N/A | What are the extent and nature of the most common long COVID symptoms by country in   2020 and 2021? | meta-analysis and systematic review | fatigue, cognitive problems, shortness of breath, memory problems, fatigue, dyspnea s | ranged up to 12 months | N/A | N/A | N/A | mild illness |
| Hazan 2022 | High | 10 | The aim of this study is to identify markers for severe acute disease and chronic long-term complications of COVID-19 in a hospitalized pediatric population | Cohort study | 12% of the symptomatic patients had chronic respiratory complications (persistent tachypnea, persistent hypoxemia, and/or chronic ground glass opacities in chest CT); non-respiratory symptoms (13%): recurrent leg pain w/ erythema, Guillain Barre, adrenal insufficiency, MIS-C | N/A | symptomatic patients who had radiologic follow up, 16.7% were found to have chronic chest radiograph abnormalities (chronic atelectesis, chronic opacities c/w scarring) | Oxygen for hypoxemic patients | pulmonary co-morbidity (associated with increased risk for chronic disease), obesity | All patients in this article were hospitalized; 66% symptomatic COVID (rest were there for things like surgery or trauma); 31% were admitted to PICU |
| Haelle 2021 | N/A | N/A | Describe prevalence of Long COVID in children | Description of a study | Respiratory symptoms in 2% of cases, systemic (such as fatigue and fever) in 2% of cases, neurologic (such as headache, seizures, and continued loss of taste or smell) in 1% of cases, and psychological (such as new-onset depression and anxiety) in 1% of cases; children who presented to the ED with at least seven symptoms were four times more likely to have long-term symptoms than those who presented with fewer symptoms (OR, 4.02; P = .01) | N/A | N/A | N/A | N/A | both severe and asymptomatic were included |
| Gupta 2022 | N/A | 373 w/ neuro involvement; 1 w. memory dificulties and agitation; 306 w/ difficulty concentrationg, 234 w/ memory problems, 204 w/ every day task challenges, 410 with fatigue | To review the empirical evidence regarding neuropsychaitric illness (Long COVID) in children and adolescents post-severe acute respiratory coronavirus infection | Systematic review | 33% of pts in US based study with neurologic or psychiatric symptoms (including: OCD symptoms, emotional lability, facial motor tics, guttural tics, aggressiveness, irritability, inattentiveness, inappetence and lack of attention); prolonged HAs, difficulty concentrating, brain fog, memory loss; GBS, encephalopathy, demyelination of CNS, stroke, and acute fulminant edema | 6 months for some | N/A | N/A | N/A | some with severe COVID-19 disease |
| Fashina 2022 **(**poster abstract extracted from COVIDENCE, J. PIDS paper 2023 publication also added) | High | 37 | Explore the clinical spectrum and out-comes of children and adolescents diagnosed with PASC within a large healthcare system | Cross sectional study | fatigue, dyspnea, HAs, anosmia, chest pain, dizziness/lightheadedness, brain fog, sleep disturbance, loss of appetite, recurrent fever, abd pain, palpitations, myalgias | N/A | N/A | N/A | atopy (32%), asthma (22%), obesity (12%); 28% w/ no past medical history | 88% w/ mild illness, 12% w/hospitalization |
| Ebell 2022 | High | 14 | To determine the prevalence of previous infection, risk factors for infection, and the prevalence of persistent symptoms following infection among university students | Cross sectional study | fatigue, exertional dyspnea, MSK pain, brain fog, anxiety | surveyed patinets up to 2 months | N/A | N/A | N/A | fever, cough, dyspnea, fatigue |
| Drogalis-Kim 2022 | High | 9 | To highlight the phenomenon of dysautonomia after acute SARS-CoV-2 infection and its response to therapy | Case series | daily dizziness, lightheadedness, unsteadiness with positional changes, fatigue, headaches, difficulty concentrating, dyspnea on exertion, nausea/vomiting/depressed appetite, anxiety |  | Orthostatic hypotension testing (Active Standing Test), EKG (all normal), cardiac monitor, cardio-pulm exercise testing, +/- laboratory testing | Non-pharmacologic therapy: 1. 2.5-3L of water per day 2. 8-10g sodium per day 3. compression garments 4. improved sleep hygiene 5. daily aerobic exercise regimen (can start with recumbent or semi-recumbent if extremely symptomatic 6. lower extremity strengthening exercises; Pharmacologic therapy: for POTS: fludrocortisone, atenolol, midodrine; Other: CBT for orthostatic intolerance , acupuncture | 3 pts w with past medical history of anxiety; 1 with occasional dizziness prior to COVID, 1 with asthma | All experienced mild acute symptoms; 6 w/ fever, 5 with URI sxs, 1 w/ GI sxs, 5 w/ anosmia or anguesia |
| Brackel 2022 | N/A | 431 | To assess the currently available pediatric international long-COVID care programs and explore the characteristics of their patient cohorts | Cohort study | fatigue, headaches, concentration difficulties, dyspnea and sleep disturbances. 5-37% of patients had severe limitations in daily life | some patients suffered from complaints for more than twelve weeks | N/A | N/A | most commonly atopic syndrome, asthma and prematurity | Most patients (90-100%) suffered from asymptomatic or mild acute COVID-19 |
| Bhargava 2021 | High | 64 | Characterize symptoms onset, symptoms, and duration of symptoms in pediatric PASC from NJ clinic | Cohort study | 55.6% experienced fatigue, 50% experienced shortness of breath or cough, 50% experienced brain fog, 33% chest pain and 44.4% experienced anxiety and/or depression | at least 4 weeks | N/A | N/A | N/A | N/A |
| Barmherzig 2021 | N/A | 17 | Describe headaches in pediatric patients following COVID-19 | Case series | Moderate to severe daily headaches(frontal or holocephalic- 94% (16/17), fatigue, decreased exercise tolerance, myalgias, cognitive complaints, dizziness, altered smell, emotional changes, poor sleep, and GI symptoms | up to 6 months | Neuroimaging following infection (n = 4), all had normal studies or chronic findings unrelated to COVID-19. | All patients tried anti-inflammatory drugs (NSAIDs) without benefit. Other treatments offered following initial presentation included oral steroids (n = 3), peripheral nerve blocks (n = 3), and Emergency Room or inpatient migraine treatments such as intravenous ketorolac,metoclopramide and methylprednisolone (n = 5). Nerve blocks were not associated with meaningful benefit in all three patients who received this intervention. Nutraceutical supplements most commonly recommended were magnesium, vitamin D, and melatonin. Preemptive treatment with zonisamide and gabapentin were associated with a more favorable response (n = 7). | Background history of episodic headaches preceding COVID infection was present in 83.3%  (15/17) of included patients | Headache |
| Chou 2022 | High | N/A | Assess whether SARS-CoV-2 is an asthma trigger resulting in poor asthma control | Cross sectional study | asthma symptoms | N/A | N/A | N/A | Asthma | N/A |
| Rao 2024 | N/A | N/A | To identify diagnosed symptoms, health conditions and medications in children with PASC. | Cohort study | loss of taste or smell, myocarditis, chest pain, hair loss, abnormal liver enzymes, skin rashes, fatigue, maliase, fever and chills, GI issues, disorders of teeth or gingiva, anxiety | N/A | N/A | N/A | N/A | N/A |
| Enner 2022 | Moderate | 47-- all with MISC related PASC | describe long term sequelae of MISC | Cohort study | neurologic symptoms, psychiatric symptoms, sleep disturbances, | N/A | N/A | N/A | N/A | all hospitalized for MIS-C |
| Messiah 2022 | High | 111 (11 from MISC Cohort and 110 from non-MISC Cohort) | Comparison of Long-Term Complications of COVID-19 Illness among a Diverse Sample of Children by MIS-C Status | Cross sectional study | Neurologic symptoms, seizure, headaches, attention difficulty, sleep symptoms, mood/ behavioral changes | N/A | N/A | N/A | N/A | 84% of patients were hospitalized with acute COVID-19 illness |

References:

1.PRESS Peer Review of Electronic Search Strategies: 2015 Guideline Statement. J Clin Epidemiol. 2016 Jul 1;75:40–6.

2.Covidence systematic review software, Veritas Health Innovation, Melbourne, Australia. Available at . Accessed August 2022.

3.Page MJ, McKenzie JE, Bossuyt PM, Boutron I, Hoffmann TC, Mulrow CD, et al. The PRISMA 2020 statement: an updated guideline for reporting systematic reviews. The BMJ [Internet]. 2021 [cited 2024 Feb 21];372. Available from: <https://www.ncbi.nlm.nih.gov/pmc/articles/PMC8005924/>

4.A.  Hayden J, Windt DA van der, L.  Cartwright J, Côté P, Bombardier C. Assessing Bias in Studies of Prognostic Factors. Ann Intern Med [Internet]. 2013 Feb 19 [cited 2024 Feb 20]; Available from: <https://www.acpjournals.org/doi/10.7326/0003-4819-158-4-201302190-00009>

5.Fashina TA, Miller CM, Paintsil E, Niccolai LM, Brandt C, Oliveira CR. Computable Clinical Phenotyping of Postacute Sequelae of COVID-19 in Pediatrics Using Real-World Data. J Pediatr Infect Dis Soc. 2023 Feb;12(2):113.

6.Maddux AB, Berbert L, Young CC, Feldstein LR, Zambrano LD, Kucukak S, Newhams MM, Miller K, FitzGerald MM, He J, Halasa NB, Cvijanovich NZ, Loftis LL, Walker TC, Schwartz SP, Gertz SJ, Tarquinio KM, Fitzgerald JC, Kong M, Schuster JE, Mack EH, Hobbs CV, Rowan CM, Staat MA, Zinter MS, Irby K, Crandall H, Flori H, Cullimore ML, Nofziger RA, Shein SL, Gaspers MG, Hume JR, Levy ER, Chen SR, Patel MM, Tenforde MW, Weller E, Campbell AP, Randolph AG; Overcoming COVID-19 Investigators. Health Impairments in Children and Adolescents After Hospitalization for Acute COVID-19 or MIS-C. Pediatrics. 2022 Sep 1;150(3):e2022057798. doi: 10.1542/peds.2022-057798. PMID: 35765138; PMCID: PMC10281852.

7.Rao S, Lee GM, Razzaghi H, Lorman V, Mejias A, Pajor NM, et al. Clinical Features and Burden of Postacute Sequelae of SARS-CoV-2 Infection in Children and Adolescents. JAMA Pediatr. 2022 Oct 1;176(10):1000–9.

8.Behnood S, Newlands F, O’Mahoney L, Ghahfarokhi MH, Muhid MZ, Dudley J, et al. Persistent symptoms are associated with long term effects of COVID-19 among children and young people: Results from a systematic review and meta-analysis of controlled studies. PLOS ONE. 2023 Dec 28;18(12):e0293600.

9.Leftin Dobkin SC, Collaco JM, McGrath-Morrow SA. Protracted respiratory findings in children post-SARS-CoV-2 infection. Pediatr Pulmonol. 2021;56(12):3682–7.

10.Hazan G, Shah MZ, Brennan S. Markers for severe disease and long-term sequelae in pediatric patients with severe acute respiratory syndrome coronavirus 2 infection. Pediatr Int Off J Jpn Pediatr Soc. 2022;64(1):e15177.

11.Palacios S, Krivchenia K, Eisner M, Young B, Ramilo O, Mejias A, Lee S, Kopp BT. Long-term pulmonary sequelae in adolescents post-SARS-CoV-2 infection. Pediatr Pulmonol. 2022 Oct;57(10):2455-2463. doi: 10.1002/ppul.26059. Epub 2022 Jul 8. PMID: 35775163; PMCID: PMC9349789.

12.Walsh-Messinger J, Manis H, Vrabec A, Sizemore Bs J, Bishof K, Debidda M, et al. The kids are not alright: A preliminary report of Post-COVID syndrome in university students. J Am Coll Health J ACH. 2021;1–7.

13.Morrow AK, Ng R, Vargas G, Jashar DT, Henning E, Stinson N, et al. Postacute/Long COVID in Pediatrics: Development of a Multidisciplinary Rehabilitation Clinic and Preliminary Case Series. Am J Phys Med Rehabil. 2021;100(12):1140–7.

14.Buonsenso D, Pujol FE, Munblit D, Pata D, McFarland S, Simpson FK. Clinical characteristics, activity levels and mental health problems in children with long coronavirus disease: a survey of 510 children. Future Microbiol. 2022 May;17(8):577-588. doi: 10.2217/fmb-2021-0285. Epub 2022 Apr 1. PMID: 35360923; PMCID: PMC9248023.

15.Ebell MH, Forgacs D, Shen Y, Ross TM, Hulme C, Bentivegna M, et al. High Prevalence of Both Previous Infection with SARS-CoV-2 and Persistent Symptoms. J Am Board Fam Med JABFM. 2022;35(3):570–8.

16.Thallapureddy K, Thallapureddy K, Zerda E, Suresh N, Kamat D, Rajasekaran K, et al. Long-Term Complications of COVID-19 Infection in Adolescents and Children. Curr Pediatr Rep. 2022;10(1):11–7.

17.Haelle T. Long COVID symptoms reported in 6% of pediatric patients. Chest Physician. 2021;16(8):14–14.

18.Simmons AC, Bonner A, Giel A, Pezzano A, Rothner AD. Probable New Daily Persistent Headache After COVID-19 in Children and Adolescents. Pediatr Neurol. 2022;132:1–3.

19.Drogalis-Kim D, Kramer C, Duran S. Ongoing Dizziness Following Acute COVID-19 Infection: A Single Center Pediatric Case Series. Pediatrics. 2022 Aug 1;150(2):e2022056860. doi: 10.1542/peds.2022-056860. PMID: 35642018.

20. American Academy of Pediatrics. Children and COVID-19: State Level Date Report. Available at: https://www.aap.org/en/pages/2019-novel-coronavirus-covid-19-infections/children-and-covid-19-state-level-data-report/. Accessed on March 6, 2024.

21.Messiah SE, Xie L, Mathew MS, Shaikh S, Veeraswamy A, Rabi A, Francis J, Lozano A, Ronquillo C, Sanchez V, He W, Weerakoon SM, Srikanth N, Borel M, Kapera O, Kahn J. Comparison of Long-Term Complications of COVID-19 Illness among a Diverse Sample of Children by MIS-C Status. Int J Environ Res Public Health. 2022 Oct 17;19(20):13382. doi: 10.3390/ijerph192013382. PMID: 36293968; PMCID: PMC9603408.

22. Nevid M, Lomas C, Rabinovitch N. M127 A MULTIFACETED APPROACH TO DIAGNOSIS AND MANAGEMENT OF POST COVID-19 ASSOCIATED DYSPNEA IN ADOLESCENTS. Ann Allergy Asthma Immunol. 2021 Nov;127(5):S88. doi: 10.1016/j.anai.2021.08.276. Epub 2021 Nov 4. PMCID: PMC8566860.

23.Fashina TA, Miller CM, Paintsil E, Niccolai LM, Brandt C, Oliveira CR. Computable Clinical Phenotyping of Postacute Sequelae of COVID-19 in Pediatrics Using Real-World Data. J Pediatric Infect Dis Soc. 2023 Feb 27;12(2):113-116. doi: 10.1093/jpids/piac132. PMID: 36548966; PMCID: PMC9969330.

24.Bhargava A, Farrand L, Zieniewicz S, Brenner DJ, Yeh TS, Hasan U. Pediatric Long Haul Post COVID-19: Is New Jersey’s Experience Different? Open Forum Infect Dis. 2021;8(SUPPL 1):S687.

25.Gupta M, Gupta N, Esang M. Long COVID in Children and Adolescents. Prim Care Companion CNS Disord. 2022 Apr 26;24(2):21r03218. doi: 10.4088/PCC.21r03218. PMID: 35486940.

26.Khan M, Dang LQ, Singh H, Dalrymple A, Miller A, Tanios A. Spectrum of SARS-CoV-2-Related Clinical Syndromes in Children: A Year in the Life. Clin Pediatr (Phila). 2022;61(2):188–93.

27. Barmherzig R, Chadehumbe M. Case series of headache in children and adolescents with COVID-19. Headache. 2021;61(SUPPL 1):167.

28. Enner S, Shah YD, Ali A, Cerise JE, Esposito J, Rubin L, Subramony A, Kothare S. Patients Diagnosed with Multisystem Inflammatory Syndrome in Children Have Persistent Neurologic, Sleep, and Psychiatric Symptoms After Hospitalization. J Child Neurol. 2022 Apr;37(5):426-433. doi: 10.1177/08830738221075924. Epub 2022 Jan 24. PMID: 35072534.

29. Lindan CE, Mankad K, Ram D, Kociolek LK, Silvera VM, Boddaert N, et al. Neuroimaging manifestations in children with SARS-CoV-2 infection: a multinational, multicentre collaborative study. Lancet Child Adolesc Health. 2021 Mar;5(3):167.

30. Malone LA, Morrow A, Chen Y, Curtis D, De Ferranti SD, Desai M, et al. Multi‐disciplinary collaborative consensus guidance statement on the assessment and treatment of p ostacute sequelae of SARS‐CoV ‐2 infection ( PASC ) in children and adolescents. PM&R. 2022 Oct;14(10):1241–69.

31.Schober ME, Robertson CL, Wainwright MS, Roa JD, Fink EL. COVID-19 and the Pediatric Nervous System: Global Collaboration to Meet a Global Need. Neurocrit Care. 2021;35(2):283–90.

32. Morrow AK, Ng R, Vargas G, Jashar DT, Henning E, Stinson N, et al. Postacute/Long COVID in Pediatrics: Development of a Multidisciplinary Rehabilitation Clinic and Preliminary Case Series. Am J Phys Med Rehabil. 2021 Dec;100(12):1140.

33. Klein J, Wood J, Jaycox JR, Dhodapkar RM, Lu P, Gehlhausen JR, et al. Distinguishing features of long COVID identified through immune profiling. Nature. 2023 Nov;623(7985):139–48.

34. Azzam A, Khaled H, Refaey N, Mohsen S, El-Emam OA, Dawood N, et al. The burden of persistent symptoms after COVID-19 (long COVID): a meta-analysis of controlled studies in children and adults. Virol J. 2024 Jan 11;21(1):16.

35. Fernandez-de-las-Peñas C, Notarte KI, Macasaet R, Velasco JV, Catahay JA, Ver AT, et al. Persistence of post-COVID symptoms in the general population two years after SARS-CoV-2 infection: A systematic review and meta-analysis. J Infect. 2024 Feb 1;88(2):77–88.

36. Lopez-Leon S, Wegman-Ostrosky T, Valle NCA del, Perelman C, Sepulveda R, Rebolledo PA, et al. Long-COVID in children and adolescents: a systematic review and meta-analyses. Sci Rep [Internet]. 2022 [cited 2024 Feb 25];12. Available from: <https://www.ncbi.nlm.nih.gov/pmc/articles/PMC9226045/>

37. Bai F, Tomasoni D, Falcinella C, Barbanotti D, Castoldi R, Mulè G, et al. Female gender is associated with long COVID syndrome: a prospective cohort study. Clin Microbiol Infect. 2022 Apr;28(4):611.e9-611.e16.

38. Goretzki SC, Brasseler M, Dogan B, Hühne T, Bernard D, Schönecker A, et al. High Prevalence of Alternative Diagnoses in Children and Adolescents with Suspected Long COVID—A Single Center Cohort Study. Viruses [Internet]. 2023 Feb [cited 2024 Feb 24];15(2). Available from: <https://www.ncbi.nlm.nih.gov/pmc/articles/PMC9961131/>

39. Brackel CLH, Noij LCE, Vijverberg SJH, Legghe CL, Maitland-van der Zee AH, van Goudoever JB, et al. International Care programs for Pediatric Post-COVID Condition (Long COVID) and the way forward. Pediatr Res. 2024 Jan 29;1–6.

40. Borch L, Holm M, Knudsen M, Ellermann-Eriksen S, Hagstroem S. Long COVID symptoms and duration in SARS-CoV-2 positive children — a nationwide cohort study. Eur J Pediatr. 2022 Apr 1;181(4):1597–607.

41. Berg SK, Palm P, Nygaard U, Bundgaard H, Petersen MNS, Rosenkilde S, et al. Long COVID symptoms in SARS-CoV-2-positive children aged 0–14 years and matched controls in Denmark (LongCOVIDKidsDK): a national, cross-sectional study. Lancet Child Adolesc Health. 2022 Sep 1;6(9):614–23.

42. Rayner DG, Wang E, Su C, Patel OD, Aleluya S, Giglia A, et al. Risk factors for long COVID in children and adolescents: a systematic review and meta-analysis. World J Pediatr. 2024 Feb 1;20(2):133–42.

43. Morello R, Mariani F, Mastrantoni L, Rose CD, Zampino G, Munblit D, et al. Risk factors for post-COVID-19 condition (Long Covid) in children: a prospective cohort study. eClinicalMedicine [Internet]. 2023 May [cited 2024 Feb 25];59. Available from: <https://www.ncbi.nlm.nih.gov/pmc/articles/PMC10101848/>

44. Buonsenso D, Morello R, Mariani F, De Rose C, Mastrantoni L, Zampino G, et al. Risk of long Covid in children infected with Omicron or pre-Omicron SARS-CoV-2 variants. Acta Paediatr. 2023 Jun 1;112(6):1284–6.

45. Yin K, Peluso MJ, Luo X, Thomas R, Shin MG, Neidleman J, et al. Long COVID manifests with T cell dysregulation, inflammation and an uncoordinated adaptive immune response to SARS-CoV-2. Nat Immunol. 2024 Feb;25(2):218–25.

46. Mizrahi B, Sudry T, Flaks-Manov N, Yehezkelli Y, Kalkstein N, Akiva P, et al. Long covid outcomes at one year after mild SARS-CoV-2 infection: nationwide cohort study. BMJ [Internet]. 2023 Jan 11 [cited 2024 Feb 25];380. Available from: <https://www.bmj.com/content/380/bmj-2022-072529>

47. Buonsenso D, Pazukhina E, Gentili C, Vetrugno L, Morello R, Zona M, et al. The Prevalence, Characteristics and Risk Factors of Persistent Symptoms in Non-Hospitalized and Hospitalized Children with SARS-CoV-2 Infection Followed-Up for up to 12 Months: A Prospective, Cohort Study in Rome, Italy. J Clin Med [Internet]. 2022 Nov [cited 2024 Feb 12];11(22). Available from: <https://www.ncbi.nlm.nih.gov/pmc/articles/PMC9692851/>

48. Dun-Dery F, Xie J, Winston K, Burstein B, Gravel J, Emsley J, et al. Post–COVID-19 Condition in Children 6 and 12 Months After Infection. JAMA Netw Open [Internet]. 2023 Dec [cited 2024 Feb 12];6(12). Available from: <https://www.ncbi.nlm.nih.gov/pmc/articles/PMC10755606/>

49. Razzaghi H, Forrest CB, Hirabayashi K, Wu Q, Allen A, Rao S, et al. Vaccine Effectiveness Against Long COVID in Children. Pediatrics [Internet]. 2024 Jan 16 [cited 2024 Feb 25]; Available from: <https://publications.aap.org/pediatrics/article/doi/10.1542/peds.2023-064446/196419/Vaccine-Effectiveness-Against-Long-COVID-in-Children>

50. Seylanova N, Chernyavskaya A, Degtyareva N, Mursalova A, Ajam A, Xiao L, et al. Core Outcome Measurement Set for Research and Clinical Practice in Post COVID-19 Condition (Long COVID) in Children and Young People: An International Delphi Consensus Study “PC-COS Children.” Eur Respir J [Internet]. 2024 Jan 1 [cited 2024 Mar 7]; Available from: <https://erj.ersjournals.com/content/early/2023/12/07/13993003.01761-2023>

51. World Health Organization. Post–COVID-19 condition (long COVID). Available at: https://www.who.int/europe/news-room/fact-sheets/ item/post-covid-19-condition#::text=Definition,months%20with% 20no%20other%20explanation. Accessed July 2023.

52. Center for Disease Control. Long COVID or Post-COVID Conditions. Available at: [https://www.cdc.gov/coronavirus/2019-ncov/long-term-effects. Accessed October 2023](https://www.cdc.gov/coronavirus/2019-ncov/long-term-effects.%20Accessed%20October%202023)

53. Dowse, Thomas Stretch. On brain and nerve exhaustion (neurasthenia) :and on the nervous sequelae of influenza. London : Baillière, Tindall and Cox, 1894.

54. Hunter C. THE LATE SEQUELAE OF ENCEPHALITIS LETHARGICA AND OF INFLUENZA. Can Med Assoc J. 1931 Jun;24(6):828-30. PMID: 20318336; PMCID: PMC382505.

55. Trojan DA, Cashman NR. Post-poliomyelitis syndrome. Muscle Nerve. 2005 Jan;31(1):6-19. doi: 10.1002/mus.20259. PMID: 15599928.

56. Delogu AB, Aliberti C, Birritella L, De Rosa G, De Rose C, Morello R, Cambise N, Marino AG, Belmusto A, Tinti L, Di Renzo A, Lanza GA, Buonsenso D. Autonomic cardiac function in children and adolescents with long COVID: a case-controlled study. Eur J Pediatr. 2024 May;183(5):2375-2382. doi: 10.1007/s00431-024-05503-9. Epub 2024 Mar 6. PMID: 38446228; PMCID: PMC11035407.

57. Baldi, Fabiana MD*; De Rose, Cristina MD†; Mariani, Francesco MD†; Morello, Rosa MD†; Raffaelli, Francesca MD‡; Valentini, Piero MD†; Buonsenso, Danilo MD†,§. Cardiopulmonary Exercise Testing in Children With Long COVID: A Case-controlled Study. The Pediatric Infectious Disease Journal ():10.1097/INF.0000000000004371, May 07, 2024. | DOI: 10.1097/INF.0000000000004371

58. Buonsenso D, Valentini P, De Rose C, Tredicine M, Pereyra Boza MDC, Camponeschi C, Morello R, Zampino G, Brooks AES, Rende M, Ria F, Sanguinetti M, Delogu G, Sali M, Di Sante G, On Behalf Of The Gemelli-Pediatric Covid-Team. Recovering or Persisting: The Immunopathological Features of SARS-CoV-2 Infection in Children. J Clin Med. 2022 Jul 27;11(15):4363. doi: 10.3390/jcm11154363. PMID: 35955979; PMCID: PMC9369242.

59. Surapaneni KM, Singhal M, Saggu SR, Bhatt A, Shunmathy P, Joshi A. A Scoping Review on Long COVID-19: Physiological and Psychological Symptoms Post-Acute, Long-Post and Persistent Post COVID-19. Healthcare (Basel). 2022 Nov 30;10(12):2418. doi: 10.3390/healthcare10122418. PMID: 36553942; PMCID: PMC9778115.

60. Choutka J, Jansari V, Hornig M, Iwasaki A. Unexplained post-acute infection syndromes. Nat Med. 2022 May;28(5):911-923. doi: 10.1038/s41591-022-01810-6. Epub 2022 May 18. Erratum in: Nat Med. 2022 Aug;28(8):1723. doi: 10.1038/s41591-022-01952-7. PMID: 35585196.
