# Supplementary material for: Postacute Sequelae of COVID-19 in Pediatric Patients Within the United States: A Scoping Review
Source: Am J Med Open. 2024 Sep 26;12:100078. doi: 10.1016/j.ajmo.2024.100078 (PMC11617896; doi:10.1016/j.ajmo.2024.100078)
Supplement: Supplementary file 2 [file mmc2.docx]

Supplemental Figure 1: Study and description of initial COVID-19 symptoms experienced by the patients

| **Study** | **Initial COVID-19 symptoms** |
| --- | --- |
| Walsh-Messinger et al 2021 | 36% mild, 59% moderate,  4.5% severe |
| Thallapureddy et al 2022 | 19.2% asymptomatic |
| Simmons et al 2022 | all mild infection |
| Palacios et al 2022 | all mild |
| Morrow et al 2021 | 1 out of the 9 patients were hospitalized for MISC, the other 8 had mild symptoms |
| Maddux et al 2022 | All patiets with acute COVID and MISC were hospitalized |
| Leftin Dobkin et al 2021, | 4/29 patients (14%) required hospitalization; rest were mild with fever, cough, dyspnea, ageusia/anosmia, myalgia; duratio n of illness was 13.4+/- 11 days; 1 patient with MISC |
| Khan et al 2022 | all mild |
| Hirt et al 2022 | 57% hospitalized |
| Hazan et al 2022 | 65% hospitalized for symptomatic COVID-19 |
| Gupta et al 2022 | al with severe disease |
| Fashina et al 2022 | 88% w/ mild illness, 12% w/hospitalization |
| Ebell et al 2022 | all mild or asymptomatic |
| Drogalis-Kim et al 2022 | All experienced mild acute symptoms |
| Brackel et al 2022 | Most patients (90-100%) suffered from  asymptomatic or mild acute COVID-19 |
| Borel et al 2022 | all mild or asymptomatic |
| Blitshteyn 2021 | 58% had a mild-tomoderate course of COVID-  19 requiring no hospitalization, 41% required hospitalization or intensive care unit admission |
| Barmherzig et al 2021 | all mild and included headache |

Supplemental Figure 2: PASC symptoms described in each study
